# Characterization of a Metal-Resistant *Bacillus* Strain With a High Molybdate Affinity ModA From Contaminated Sediments at the Oak Ridge Reservation

**DOI:** 10.3389/fmicb.2020.587127

**Published:** 2020-10-19

**Authors:** Xiaoxuan Ge, Michael P. Thorgersen, Farris L. Poole, Adam M. Deutschbauer, John-Marc Chandonia, Pavel S. Novichkov, Sara Gushgari-Doyle, Lauren M. Lui, Torben Nielsen, Romy Chakraborty, Paul D. Adams, Adam P. Arkin, Terry C. Hazen, Michael W. W. Adams

**Affiliations:** ^1^Department of Biochemistry and Molecular Biology, University of Georgia, Athens, GA, United States; ^2^Environmental Genomics and Systems Biology Division, Lawrence Berkeley National Laboratory, Berkeley, CA, United States; ^3^Earth and Environmental Sciences, Lawrence Berkeley National Laboratory, Berkeley, CA, United States; ^4^Molecular Biosciences and Integrated Bioimaging, Lawrence Berkeley National Laboratory, Berkeley, CA, United States; ^5^Department of Bioengineering, University of California, Berkeley, Berkeley, CA, United States; ^6^Department of Civil and Environmental Engineering, The University of Tennessee, Knoxville, Knoxville, TN, United States

**Keywords:** *Bacillus* sp. XG196, nitrate, nitrate reductase, molybdenum limitation, molybdate transport

## Abstract

A nitrate- and metal-contaminated site at the Oak Ridge Reservation (ORR) was previously shown to contain the metal molybdenum (Mo) at picomolar concentrations. This potentially limits microbial nitrate reduction, as Mo is required by the enzyme nitrate reductase, which catalyzes the first step of nitrate removal. Enrichment for anaerobic nitrate-reducing microbes from contaminated sediment at the ORR yielded *Bacillus* strain EB106-08-02-XG196. This bacterium grows in the presence of multiple metals (Cd, Ni, Cu, Co, Mn, and U) but also exhibits better growth compared to control strains, including *Pseudomonas fluorescens* N2E2 isolated from a pristine ORR environment under low molybdate concentrations (<1 nM). Molybdate is taken up by the molybdate binding protein, ModA, of the molybdate ATP-binding cassette transporter. ModA of XG196 is phylogenetically distinct from those of other characterized ModA proteins. The genes encoding ModA from XG196, *P. fluorescens* N2E2 and *Escherichia coli* K12 were expressed in *E. coli* and the recombinant proteins were purified. Isothermal titration calorimetry analysis showed that XG196 ModA has a higher affinity for molybdate than other ModA proteins with a molybdate binding constant (K_*D*_) of 2.2 nM, about one order of magnitude lower than those of *P. fluorescens* N2E2 (27.0 nM) and *E. coli* K12 (25.0 nM). XG196 ModA also showed a fivefold higher affinity for molybdate than for tungstate (11 nM), whereas the ModA proteins from *P. fluorescens* N2E2 [K_*D*_ (Mo) 27.0 nM, K_*D*_ (W) 26.7 nM] and *E. coli* K12[(K_*D*_ (Mo) 25.0 nM, K_*D*_ (W) 23.8 nM] had similar affinities for the two oxyanions. We propose that high molybdate affinity coupled with resistance to multiple metals gives strain XG196 a competitive advantage in Mo-limited environments contaminated with high concentrations of metals and nitrate, as found at ORR.

## Introduction

Molybdenum (Mo) is an essential metal for the growth of virtually all known life forms, including humans, plants and microorganisms, as it is required for the function of several key enzymes involved in the cycling of N, C, and S (Hamlin, 2016; Schwarz, 2016; Maia et al., 2017). Tungsten (W), an antagonist of Mo, is more uncommon in nature but required in some enzymes, most notably in archaea. Physiologically-relevant oxidation states of Mo and W are + 4, + 5, and + 6 (Maia et al., 2017). There are five distinct enzyme families that use Mo and/or W, represented by nitrogenase (Mo only, although some use vanadium), xanthine oxidase (Mo only), the sulfite oxidase (Mo only), DMSO reductase (most family members use Mo, a few use W) and tungsten-containing oxidoreductase (WOR, W only) (Hille et al., 2014; Maia et al., 2017). In most microorganisms, molybdate is taken up into the cell by the molybdate ATP-binding cassette or Mod transporter (ModABC), which can also take up tungstate (Grunden and Shanmugam, 1997; Self et al., 2001).

In the nitrogen cycle, Mo is utilized in three key steps, N_2_-fixation (by nitrogenase), nitrite oxidation (by nitrite oxidoreductase) and nitrate reduction (by nitrate reductase) (Zhang and Gladyshev, 2008). Hence Mo is required for the biological removal of nitrate from contaminated environments as the reductase is a key enzyme in both the denitrification (yielding N_2_) and dissimilatory nitrate reduction to ammonium (DNRA) pathways (Zhang and Gladyshev, 2008). Consequently, in natural environments, the availability of Mo can limit nitrate removal (Barron et al., 2009; Glass et al., 2012). Mo limitation can also negatively impact nitrate removal in contaminated environments, which can be caused by the extensive use of nitrate-containing fertilizers, the release of nitrate-containing industrial wastes, as well as mining and other anthropogenic activities leading to problems for human health and natural environments (Spalding and Exner, 1993; Kellman and Hillaire-Marcel, 2003; Diaz and Rosenberg, 2008; Gruber and Galloway, 2008; Powlson et al., 2008; Thorgersen et al., 2015; Zhang et al., 2015).

The Oak Ridge Reservation (ORR) in Tennessee, United States contains a nitrate-contaminated waste site – the S-3 ponds. These are four adjacent (∼9.5 million liters each) earthen reservoirs used for the disposal of waste liquids that had been produced from the Y-12 nuclear plant for more than 30 years (Brooks, 2001). The waste liquids contained high, and potentially toxic, concentrations of nitrate (up to 1.2 M) and a wide variety of metals, such as iron (up to 21 mM), aluminum (up to 180 mM), magnesium (up to 28 mM), and uranium (up 1.3 mM) (Brooks, 2001). In 1983, the waste liquids in the S-3 ponds were adjusted to about pH 9, and the precipitates formed were allowed to settle before the liquid was removed (Brooks, 2001; Revil et al., 2013). In 1988, the S-3 ponds were filled and capped and now serve as a parking lot (Revil et al., 2013). However, the area is still heavily contaminated and groundwater in the contamination plume emanating from the former S-3 ponds is at low pH (as low as 3.0) and contains high concentrations of nitrate (up to 230 mM), much higher than the surrounding pristine groundwater (less than 32 μM) (Nolan et al., 1998; Ge et al., 2019). In addition, the contaminating plume has elevated concentrations of over 20 metals, including uranium (up to 580 μM) (Smith et al., 2015; Thorgersen et al., 2015). In stark contrast, extremely low concentrations of Mo (in the picomolar range) were measured in this highly contaminated groundwater. It was demonstrated experimentally that the pM concentrations of Mo in ORR contaminated groundwater were likely a result of molybdate adsorption and incorporation into Fe- and Al-based minerals that are formed as the groundwater from the highly contaminated area (pH < 1) mixes with the surrounding groundwater (Moura et al., 2004; Ge et al., 2019).

Hence, a fundamental question is whether microorganisms that thrive in the unique ORR environment contaminated with high concentrations of metals and nitrate, yet containing only picomolar levels of Mo, have unique features that enhance Mo utilization. Herein, we describe the characterization of a novel nitrate-reducing *Bacillus*, designated strain EB106-08-02-XG196 (hereafter XG196), that was isolated from a sample of nitrate- and metal-contaminated ORR sediment (EB-106) located 21 m downstream of the S-3 ponds area (Ge et al., 2019). XG196 is resistant to high concentrations of a metal mixture that was designed to mimic the ORR contaminated groundwater. More importantly, it is also much less sensitive to Mo-limitation than other ORR isolates, including four other EB-106 strains and a microbe obtained from a non-contaminated ORR environment. The molecular basis for the ability of XG196 to thrive under Mo-limited conditions was investigated.

## Materials and Methods

### Sampling and Isolation of Strains

An 8-meter-deep borehole of 8.9 cm diameter (designated EB-106) located 21.1 meters downstream from the S-3 ponds area was drilled at ORR. The sediment was collected and cut into 22 cm segments under anaerobic conditions, as reported elsewhere (Ge et al., 2019; Moon et al., 2020). For microbial enrichment, sediment samples (1 g) were incubated anaerobically in 5 ml of a defined medium (pH 7.0) containing 1.3 mM KCl, 2 mM MgSO_4_, 0.1 mM CaCl_2_, 0.3 mM NaCl, 30 mM NaHCO_3_, 5 mM NaH_2_PO_4_ and 20 mM NaNO_3_, with added vitamins and minerals as described (Widdel and Bak, 1992). A mixture of 2 mM of different carbon sources (formate, acetate, ethanol, lactate, succinate, and glucose) and 0.1 g/L yeast extract was used as carbon sources. Metal mix (MM) was used to mimic the metal contamination in the groundwater near the ORR S-3 ponds. MM (1x) resulted in final concentrations in the media of 5 μM cadmium acetate (Cd(CH_3_COO)_2_⋅2H_2_O), 100 μM manganous chloride (MnCl_2_⋅2H_2_O), 30 μM cobalt chloride (CoCl_2_⋅6H_2_O), 100 μM nickel chloride (NiCl_2_⋅6H_2_O), 10 μM cupric chloride (CuCl_2_⋅2H_2_O), 10 μM ferrous ammonium sulfate (Fe(NH_4_)_2_(SO_4_)_2_ ⋅6H_2_O) and 100 μM uranyl acetate (UO_2_(CH_3_COO)_2_⋅2H_2_O) (Supplementary Table S1). For enrichment, all cultures were incubated anaerobically in anaerobic chamber (filled with 95% argon and 5% hydrogen) at room temperature for 2 to 7 days in media containing either 1 ×, 0.5 × or no MM of final concentrations. Cultures with turbidity were streaked out on plates (1.5% agar) using the same medium and were incubated at room temperature for single colony isolation. For purified microbial strains, the sequences of their 16S rRNA genes were determined as described below and compared with those in the BLASTN suite^1^ using default parameters (Altschul et al., 1990).

### Nitrate Reductase Activity

Nitrate reductase activities of the EB-106 isolates were determined using whole cell suspensions (Filiatrault et al., 2013). Strains were grown anaerobically in Hungate tubes and cells were collected between mid-log phase and early stationary phase, then 15 μL of 5 mg/ml chloramphenicol was added to 1.5 ml of culture to inhibit protein synthesis. Cells were washed twice and re-suspended in buffer (50 mM phosphate buffer, pH 7.2) and the OD_660_ was determined. 200 μL of cells were mixed with 25 μL of methyl viologen (0.5 mg/ml) in an anaerobic sealed cuvette at 25°C. 100 μL of reaction solution (4 mg/ml Na_2_S_2_O_4_, 4 mg/ml NaHCO_3_ and 100 mM KNO_3_) was added to start the reaction. In control reaction buffer, Na_2_S_2_O_4_ was replaced with water. After incubation at room temperature for 5 min, the mixtures were vortexed in air to stop the reaction by oxidizing the electron donors (Na_2_S_2_O_4_ and reduced methyl viologen). The amount of nitrite produced was measured by adding 100 μL of sulfanilic acid (1% w/v in 20% HCl) to 30 μL of each reaction mixture followed by 100 μL of *N*-(1-naphthyl)ethylenediamine-HCl (1.3 mg/ml). The OD_540_ of each sample supernatant was measured and the amount of nitrite was calculated according to nitrite standards. The OD_420_ of the samples was also measured to account for light scattering by residual cells and cell fragments. Nitrate reductase specific activity is expressed as units/OD_660_, in which units are calculated using the formula 100 × [OD_540_ - (0.72 × OD_420_)]/(T × V), T is time in minutes and V is reaction volume in milliliters (Filiatrault et al., 2013; Thorgersen et al., 2019).

### Carbon Sources Utilization for Anaerobic Growth Analysis

Growth on various carbon sources was determined at 25°C under anaerobic conditions using the standard medium lacking yeast extract and the organic mixture but containing either formate, acetate, ethanol, lactate, succinic acid, fumarate, xylose, xylitol, glucose, fructose, maltose, sodium benzoate, sodium 4-hydroxybenzoate, potassium sodium tartrate, proline, phenylalanine, arginine, threonine, leucine, glutamate, or glutamine (all at 2 mM) with and without nitrate (KNO_3_, 20 mM). Growth was measured in 400 μl wells on a 100-well plate (Bioscreen sterile plates HONEYCOMB, Thermo Fisher Scientific, Waltham, MA, United States) using a Bioscreen C (Thermo Labsystems, Thermo Fisher Scientific, Waltham, MA, United States) placed in a PLAS LABS anaerobic chamber under a 5% H_2_ and 95% Ar atmosphere. Optical density (OD_600_) of cultures in each wells were measured every 5 min, after the plate was shaken using the Bioscreen C to resuspend cells.

### Mo Accumulation Analysis

EB-106 isolates were grown in 500 ml of defined media with 1 μM Mo ((NH_4_)_2_MoO_4_) and harvested at mid log phase, washed three times with 10 ml of Tris buffer (Tris 50 mM, pH 8.0, containing 100 mM NaCl) and then resuspended in Tris buffer. Cells were lysed by sonication, then were spun down at 10,000 × G for 15 min and the supernatants were used for further centrifugation. The cytoplasmic extract (S100) was obtained after centrifugation at 100,000 × G for 1 h in a Beckman Coulter Optima L-90 ultracentrifuge. The membrane fraction was resuspended in 2 ml of Tris buffer. Both S100 and membrane fractions were diluted (1:40) with trace grade 2% nitric acid (VWR, Radnor, PA, United States) and incubated overnight prior to analysis by inductively coupled plasma mass spectrometry (ICP-MS) analysis to quantify Mo (Lancaster et al., 2014; Scott et al., 2015). Protein concentrations were measured using the Bradford assay (Bio-Rad protein assay kit, Bio-Rad, Berkeley, CA, United States). The amount of Mo accumulated is expressed as nmoles per gram of protein (nmol/g).

### Molybdenum-Limited Growth

For the Mo-depleted medium, a solution was prepared that contained 1.3 mM KCl, 2 mM MgSO_4_, 0.1 mM CaCl_2_, and 0.3 mM NaCl together with the vitamins and minerals described above except that molybdenum and tungsten were not added (Widdel and Bak, 1992). Fe(NO_3_)_3_ (20 mM) was then added, which acidifies the solution to pH ∼ 2.5. The pH was then adjusted to pH 6.7 using trace grade NaOH (2.0 M) to induce precipitation of ferric hydroxide. As previously described (Ge et al., 2019), the Fe precipitates any contaminating Mo present in the medium components. The Mo-depleted growth medium was prepared by adding trace grade Fe(NO_3_)_3_ (7.4 μM), Na_2_SO_4_ (2 mM), NaHCO_3_ (30 mM), and NaH_2_PO_4_ (5 mM) and inoculated with 1% (vol/vol) washed XG77, XG146, XG95, XG201, or XG196 cells grown in media with no Mo added. Growth in this medium with and without added Mo (0.1, 0.5, 1, 5, 10, 50, or 500 nM Na_2_MoO_4_) was measured in quadruplet using the Bioscreen C described above. Mo and W competition analysis of XG196 and *Pseudomonas fluorescens* N2E2 under nitrate reducing conditions were performed using the same media with added Mo (0, 5, 50, 500, 5000, or 50000 nM Na_2_MoO_4_) and added W (0, 50 nM, 5 or 500 μM Na_2_WO_4_).

### Metal Tolerance Assay

Each EB-106 isolate (XG77, XG146, XG95, XG201, and XG196) was incubated with individual metals at multiple concentrations, including manganous chloride (MnCl_2_⋅2H_2_O, 0 ∼ 6 mM), cobalt chloride (CoCl_2_⋅6H_2_O, 0∼600 μM), nickel chloride (NiCl_2_⋅6H_2_O, 0∼250 μM), cupric chloride (CuCl_2_⋅2H_2_O, 0∼250 μM), cadmium acetate Cd(CH_3_COO)_2_⋅2H_2_O, 0∼200 μM) or uranyl acetate (UO_2_(CH_3_COO)_2_⋅2H_2_O, 0∼2 mM), or with the MM (0∼2×) described above (Supplementary Table S1). Cultures were grown in defined medium described above containing 0.1 g/L yeast extract using the Bioscreen C described above and growth data were analyzed using R with Grofit package (Kahm et al., 2010). The average half maximal inhibitory concentration (IC_50_) was used to reflect the tolerance of each EB-106 isolate to each individual metal and the MM.

### Genome Sequencing

The ZymoBead Genomic DNA kit was used to extract genomic DNA from strain XG196 and XG77. More than 1 μg of purified gnomic DNA from each strain was used for Illumina sequencing. The Illumina sequencing reads were trimmed using Trimmomatic 0.36, with parameters “-phred33 LEADING:3 TRAILING:3 SLIDINGWINDOW:4:15 MINLEN:36 ILLUMINACLIP:TruSeq3-PE.fa” (Bolger et al., 2014). The trimmed reads were assembled *de novo* using SPAdes v3.12.0 with parameters “-k 21,33,55,77” (Bankevich et al., 2012). Genes were identified using Prokka v1.12, with default parameters (Seemann, 2014). This pipeline was executed using the Department of Energy KnowledgeBase software platform (KBase^2^; Arkin et al., 2018). The genome of XG196 was submitted to GenBank (accession number: JABWSY000000000).

### 16S rDNA Sequencing and Phylogenetic Analysis

The 16S rDNA of isolate XG196 was amplified by PCR using universal bacterial primers 27F (5′-AGA GTT TGA TCC TGG CTC AG-3′) and 1492R (5′-ACG GCT ACC TTG TTA CGA CTT-3′) from Integrated DNA Technologies, Coralville, IA, United States. DNA sequencing was carried out by GENEWIZ, South Plainfield, NJ, United States. The sequence was first analyzed by BLAST^3^ (Altschul et al., 1990), which indicated that XG196 is a *Bacillus* strain. The sequence was uploaded to the Ribosomal Database Project (RDP^4^) (Cole et al., 2013). The RDP tool Seqmatch was run to find the closest relatives of isolate XG196 and the *Bacillus* type strains with high quality 16S rDNA sequences (>1200 bp). The two closest relatives of XG196 and a total of 187 *Bacillus* type strains with one out group strain were selected to build the 16S rRNA phylogenetic tree by IQ-TREE using maximum likelihood (^5^ Nguyen et al., 2014). GTR + F + R6 model was selected by ModelFinder (Kalyaanamoorthy et al., 2017) and 1000 times of bootstrapping was run using UFBoot (Hoang et al., 2017).

### Phylogenetic Analysis of Molybdate and Tungstate Binding Proteins

Accession numbers of ModA (family IPR005950) and WtpA (family IPR022498) were downloaded from the InterPro database^6^ (Mitchell et al., 2018). Information, such as sequence, mass, protein name, gene name, taxonomic lineage, cross-reference in PDB, cross-reference in KEGG, PubMed ID, etc. were all downloaded together. Proteins with candidadus/candidate organisms, uncultured organisms, fragment proteins, wrong/poorly-labeled organisms, and duplicates were removed from the list. Two lists (A and B) of strains were selected from downloaded candidates for ModA phylogenetic analysis. List A uses strains with ATCC (American Type Culture Collection) or DSM (Deutsche Sammlung von Mikroorganismen und Zellkulturen GmbH) reference IDs. For list B, all downloaded protein sequences were clustered by CD-HIT^7^ at 60% sequence identity (Huang et al., 2010). In both lists, all archaea, eukaryote sequences and sequences with KEGG cross-reference or 3D structures were kept. Several *Bacillus* type-strains and the top two closest isolate XG196 ModA relatives and ORR isolate *P. fluorescens* N2E2 from non-contaminated area were also kept. ModA/WtpA sequences from list A (617 sequences, Supplementary Material list A) and B (4623 sequences, Supplementary Material list B) were all used for tree building. Multiple sequence alignment was done by Clustal Omega^8^ (Madeira et al., 2019). IQ-tree were used to build the phylogenetic tree by maximum likelihood (Nguyen et al., 2014). LG + F + R10 model and WAG + R9 model were selected for list A and B ModA tree building by ModelFinder (Kalyaanamoorthy et al., 2017). 2000 and 3000 times of bootstrapping was run for list A and B ModA tree using UFBoot (Hoang et al., 2017). Signal peptide prediction analysis was performed for all list A sequences by SignalP5.0^9^ (Armenteros et al., 2019).

### Multi-Alignment and Structural Modeling Analysis of ModA

Multiple sequence alignments of XG196 ModA and selected proteins were first run by Clustal Omega^10^ (Madeira et al., 2019) and further analyzed with selected ModA proteins with structural data from the PDB^11^ using ESPript 3.0^12^ (Robert and Gouet, 2014). The structures of the ModA proteins from *Pyrococcus furiosus* ATCC 43587 ModA (PDB: 3CG1) and *Escherichia coli* K12 ModA (PDB: 1AMF) were used for comparison with XG196 ModA. Mean identity and mean similarity of protein sequences were also calculated by ESPript 3.0. SWISS-MODEL^13^ (Brooks, 2001) was used to predict the model of XG196 using template ModA (PDB: 2H5Y) from *Xanthomonas axonopodis* pv. *citri* 306. UCSF Chimera^14^ (Pettersen et al., 2004) was used to visualize the model.

### Expression and Purification of Recombinant ModA Proteins

ModA genes were amplified by PCR from the genomes of XG196, *P. fluorescens* N2E2 and *E. coli* K12. The primers are listed in Supplementary Table S2. The forward primer for the ModA gene of XG196 was designed to omit the N-terminal 20 amino acids, which include a signal peptide and a putative lipoprotein-attachment sight (Cys20). The forward primers for ModA genes of *P. fluorescens* N2E2 and *E. coli* K12 were designed to omit the N-terminal signal peptides, the first 23 and 25 amino acids, respectively. Signal sequences and lipoprotein-attachment site were predicted by SignalP-5.0^15^ (Armenteros et al., 2019). The PCR amplicons were cloned into the pET24a (+) plasmid (Novagen). ModA proteins were expressed in *E. coli* Rosetta 2 (DE3)pLysS (Novagen) cells in LB media supplemented with kanamycin (50 μg/ml). Recombinant gene expression was induced at an OD_600_ ∼ 0.6 with 0.5 mM IPTG and the growth temperature was reduced from 37 to 25°C. Cells were harvested after 16 h and resuspended in start buffer (Tris 20 mM, pH 7.6, 100 mM NaCl, 5 mM imidazole). Cells were lysed by sonication and centrifuged to remove unlysed cells. The supernatant fractions were loaded onto a HisTrap FF crude column (GE health care) pre-equilibrated with start buffer and washed with two column volumes of wash buffer (Tris 20 mM, pH 7.6, 100 mM NaCl, 30 mM imidazole) and the recombinant ModA proteins were then eluted with elution buffer (Tris 20 mM, pH 7.6, 100 mM NaCl, 300 mM imidazole). ModA proteins were further purified by gel filtration using a Superdex 200 HiLoad 16/60 prep grade column (GE health care) equilibrated with Tris 20 mM, pH 7.6, containing 250 mM NaCl. Fractions containing the purified ModA protein as determined by SDS-PAGE were buffer exchanged to a low salt buffer (Tris 20 mM, pH 7.6, 90 mM NaCl) using an Amicon Ultra-15 10K centrifugal filter device at 4°C for 16 h for further ITC analysis. Mo in 40 μM of protein samples before and after dialysis were measured by ICP-MS. Trace grade of Tris (MilliporeSigma, St. Louis, MO, United States) and NaCl (MilliporeSigma, St. Louis, MO, United States) were used in protein purification and dialysis.

### Isothermal Titration Calorimetry (ITC) Analysis

Molybdate (100 mM Na_2_MoO_4_) and tungstate (100 mM Na_2_WO_4_) stock solutions were prepared in trace grade ITC buffer (Tris 20 mM, pH 7.6, 90 mM NaCl) and then diluted to a final concentration of 0.3 or 0.4 mM using ITC buffer. ITC analysis was performed using a Malvern MicroCal PEAQ-ITC (Malvern Panalytical, Malvern, United Kingdom) at 25°C. Molybdate or tungstate were injected into the sample chamber (300 μL) containing 30 or 40 μM ModA to give a final molar ratio of oxyanion to ModA of 2:1. Displacement titrations were carried out by titrating molybdate or tungstate with chromate-saturated ModA (containing twofold of chromate from Na_2_CrO_4_) (Sigurskjold, 2000). Data were analyzed by Malvern MicroCal PEAQ-ITC analysis software (Malvern Panalytical, Malvern, United Kingdom). Each test was done twice and the average data were used.

### ICP-MS Analysis

Samples were vortexed and then diluted (at various concentrations depending on sample type) into 2% (vol/wt) trace-grade nitric acid (VWR, Radnor, PA, United States) in acid-washed 15 mL polypropylene tubes. Samples were analyzed by an Agilent 7900 ICP-MS fitted with MicroMist nebulizer, UHMI-spray chamber, Pt cones, x-lens and an octopole reaction system (ORS) collision cell with He-mode (Agilent Technologies, Santa Clara, CA, United States) as described (Ge et al., 2019).

### Metagenome Annotation and Analysis of Nitrate-Reducing Bacteria in ORR

Previously published metagenome sequence reads of samples from ORR groundwater were obtained from the NCBI database under BioProject PRJNA513876 (Tian et al., 2020). Metagenomic reads were preprocessed using BBtools version 38.60 (no references known^16^) to remove Illumina adapters, perform quality filtering and trimming, and remove PhiX174 spike-ins. The script bbduk.sh was run with parameters *ktrim* = *r k* = *23 mink* = *11 hdist* = *1 ref* = *adapters.fa tbo tpe 2* to remove any remaining standard Illumina adapters given in adapters.fa. The script was run again with parameters *bf1 k* = *27 hdist* = *1 qtrim* = *rl trimq* = *17 cardinality* = *t ref* = *phix174_Illumina.fa* to perform quality filtering and trimming, and to remove Illumina PhiX174 spike ins given in the file phix174_Illumina.fa. We assembled the reads using SPAdes version 3.13.0 (Bankevich et al., 2012) with parameters –*meta -k 21,33,55,77,99,127*. We predicted protein-coding genes using Prodigal v 2.6.3 (Hyatt et al., 2010) with parameters *-n -p single*. Predicted protein-coding genes were annotated on the contigs using eggNOG mapper (v2) with default parameters (Huerta-Cepas et al., 2017). The number of predicted genes for each protein of interest was normalized by the number of raw reads obtained from metagenome sequencing.

## Results

### Isolation and Physiological Characterization of *Bacillus* Strain XG196

**FIGURE 1:**
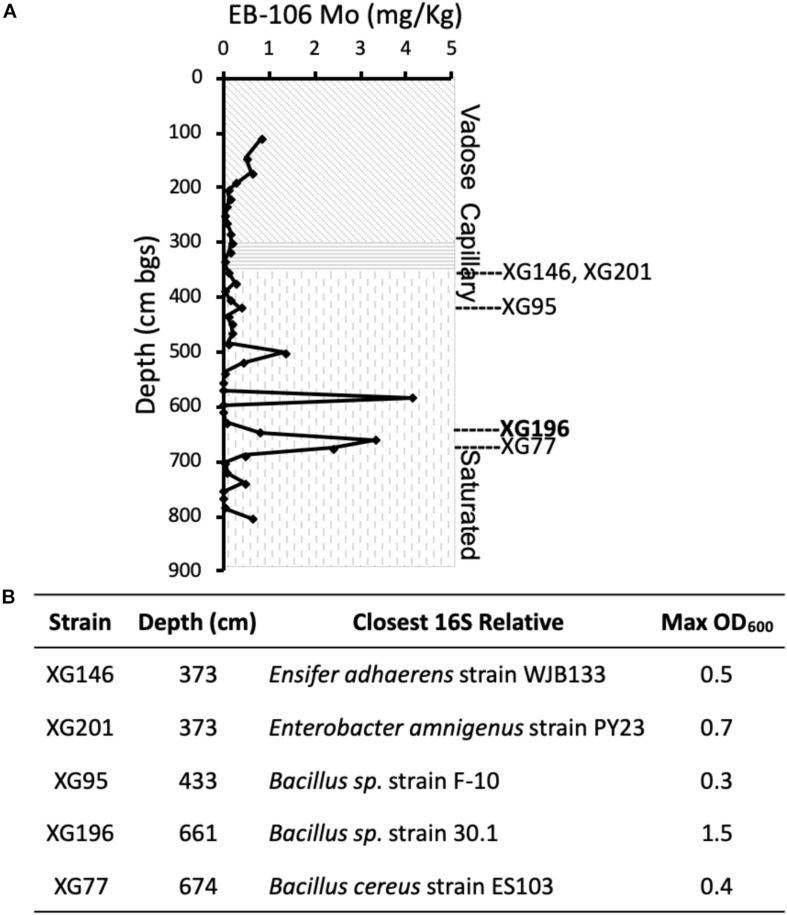
Five EB-106 sediment isolates used in this study. **(A)** Sketch of core EB-106. Subsurface layers: vadose zone (diagonal), capillary zone (horizontal), and saturated zone (vertical). Each point represents average Mo concentration at different depths. Depths of EB-106 sediments where XG146, XG201, XG95, XG196, and XG77 were isolated are indicated. **(B)** Properties of the ORR strains. Max OD_600_ was determined under nitrate reducing conditions with 20 mM carbon sources (lactate for XG196 and xylose for the other strains).

In order to isolate nitrate-reducing microbes with a high affinity for molybdate from the metal- and nitrate-contaminated ORR site, sediments from the contaminated EB-106 vertical core were used for enrichment and isolation. This 8-m core, taken about 21 m downstream of the contamination source (the S-3 ponds), was cut into 22 cm segments under anaerobic conditions (Ge et al., 2019; Moon et al., 2020). The EB-106 core covered the vadose zone (0–300 cm, the area between the land surface and water table), the capillary fringe (300–350 cm, the subsurface layer between vadose zone and the water table), and saturated zone (350–800 cm, the region below the water table) of the soil (Figure 1A). The groundwater passing through the saturated zone of the EB-106 core flows from the contamination site and is considered to be highly contaminated. A total of 88 unique nitrate-reducing bacteria were isolated from EB-106 sediment samples under nitrate-reducing conditions in a medium containing a combination of carbon sources (2 mM of formate, acetate, ethanol, lactate, succinate and glucose, together with 0.1 g/L yeast extract) and various levels of metal contaminants (no, 0.5 × MM or 1.0 × MM). Five strains, XG77, XG95, XG146, XG196 and XG201, were selected for further characterization based on their ability to grow anaerobically on nitrate, their nitrate reductase activities and metal resistance properties. XG77, XG95, and XG196 were identified as *Bacillus* strains, while XG146 and XG201 were identified as *Ensifer* and *Enterobacter* strains, respectively, by 16S rDNA sequences (Figure 1B). All five were isolated from the contaminated saturated zone (below 350 cm) (Figures 1A,B).

As shown in Supplementary Table S3, all five isolates use various carbon sources (2 mM) for nitrate-reducing growth. Isolate XG196 exhibited more robust growth on maltose (OD_600__*max*_ = 0.95), glucose (0.63), xylose (0.48), fructose (0.48), proline (0.47), glutamate (0.47), lactate (0.33), arginine (0.29) and fumarate (0.25), than other tested carbon sources (formate, acetate, ethanol, succinic acid, xylitol, benzoate, 4-hydeoxybenzoate, tartrate, phenylalanine, threonine, leucine and glutamine, OD_600__*max*_ ≤ 0.11). Xylose, glucose, fructose, maltose, proline, and glutamine also support the growth of isolate XG196 by fermentation. Lactate was selected as the carbon source for further characterization of strain XG196, as it supported robust nitrate-reducing growth and did not support fermentative growth. Xylose was selected for the other four EB-106 isolates as they exhibited good growth (XG77, OD_600__*max*_ 0.22; XG95, 0.22; XG146, 0.20; and XG201, 0.18) on xylose under nitrate-reducing conditions but did not use xylose for fermentation. Higher biomass yields were obtained with 20 mM of the preferred carbon source (lactate for isolate XG196 or xylose for strains XG146, XG201, XG95, and XG77). The maximum OD_600_ values for XG146, XG201, XG95, and XG77 increased from 0.20, 0.18, 0.22 and 0.22 to 0.5, 0.7, 0.25, and 0.4 respectively. XG196 reached the highest cell density under nitrate-reducing conditions, with OD_600_ reaching 1.5 when lactate was increased to 20 mM (Figure 1B). Nitrate reductase activities of XG146, XG201, XG95, XG77 and XG196 were determined using whole cell suspensions from cultures collected under anaerobic nitrate-reducing conditions using 20 mM nitrate (MacGregor et al., 1974; Filiatrault et al., 2013). Strains XG77 and XG196 showed higher nitrate reductase activities than the other three isolates (Supplementary Figure S1).

Growth of the EB-106 isolates was determined under nitrate-reducing growth conditions in the presence of increasing concentrations of a single metal (Cd, Ni, Cu, Co, Mn, or U) or the MM metal mixture containing all six metals, which mimics the concentrations of metals found in the ORR contaminated groundwater (Supplementary Table S1). The effects of the metals on growth was determined by calculating the IC_50_ values. Generally, strain XG196 had the highest metal tolerance of the five stains to the metal contaminants in the EB-106 sediments. Specifically, isolate XG196 had the highest IC_50_ values when grown with Ni^2+^ (119 μM), Co^2+^ (220 μM), Mn^2+^ (>900 μM), U^6+^ (2,000 μM) and the metal mixture (1.2×) and the second highest IC_50_ value when grown with Cu^2+^ (94 μM) (Supplementary Figure S2). Strain XG196 also grew in the presence of very high concentrations of nitrate and nitrite, with IC_50_ values of 299 and 99 mM, respectively (Supplementary Figure S2).

**FIGURE 2:**
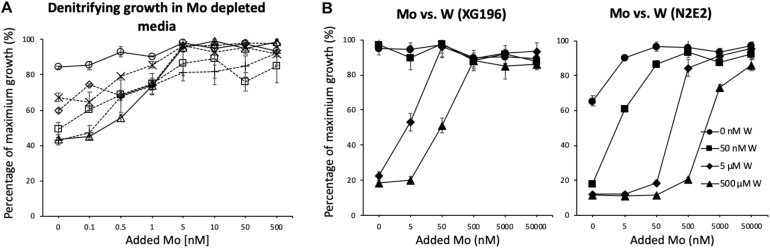
Strain XG196 is resistant to Mo limiting growth conditions. **(A)** Percentage of maximum growth (OD_600_) of ORR strains XG196 (*Bacillus*, circle), XG77 (*Bacillus*, cross), XG201 (*Enterobacter*, diamond), XG95 (*Bacillus*, square), XG146 (*Ensifer*, plus sign), and *Pseudomonas fluorescens* N2E2 (triangle) under nitrate-reducing conditions in Mo depleted media with the indicated concentration of added Mo. **(B)** Percentage of maximum growth of XG196 (left) and *P. fluorescens* N2E2 (right) under nitrate reducing conditions with tungstate at the indicated concentration competing with molybdate.

To analyze the dependence of growth under nitrate-reducing conditions on Mo, the five EB-106 strains and one strain previously isolated from non-contaminated ORR groundwater (*P. fluorescens* N2E2) were grown with increasing concentrations of molybdate in Mo depleted media prepared with trace metal grade chemicals in order to lower the amount of contaminating Mo to picomolar concentrations in cultures (∼400 pM, Ge et al., 2019). As shown in Figure 2A, strain XG196 showed the highest percentage of maximum growth (84% of highest OD_600_) even when no Mo was added to the medium, while the other EB-106 sediment strains tested required at least 1 nM Mo to reach ≥ 74% of maximum growth. *P*. *fluorescens* N2E2, which has been used as a reference strain in other ORR contamination studies (Thorgersen et al., 2015; Ge et al., 2019), had the lowest percentage (as low as 43%) of maximum growth when less than 1 nM Mo was added.

Tungstate is a competitive inhibitor of molybdate transport (Grunden and Shanmugam, 1997; Hu et al., 1997; Self et al., 2001). A Mo/W competitive growth analysis of isolate XG196 and *P*. *fluorescens* N2E2 under nitrate reducing conditions showed that low concentrations of W (up to 50 nM) do not affect the nitrate-dependent growth of XG196 but limits the growth of *P*. *fluorescens* N2E2 to only 20% of the maximum (Figure 2B). At higher W concentrations, W inhibits nitrate-dependent growth of both isolate XG196 and *P*. *fluorescens* N2E2. However, strain XG196 requires the addition of less Mo to resume maximal growth. For example, when 5 μM W was added to their media, XG196 only required 50 nM Mo to reach maximum growth, but *P*. *fluorescens* N2E2 required at least two orders of magnitude more Mo (5,000 nM; Figure 2B). Our hypothesis is that XG196 has a much higher affinity for molybdate than the other strains tested, especially that of strain N2E2. The environment from which strain N2E2 was isolated has much higher molybdate concentrations (approximately 10 nM) than the contaminated groundwater (Mo < 1 nM) (Smith et al., 2015; Thorgersen et al., 2015). A higher affinity for molybdate could give a growth advantage to XG196 by nitrate reduction under Mo-limited conditions.

### Genomic and 16S rDNA Analysis of XG196

The draft genome of strain XG196 contained 6,010,169 bp in 55 contigs longer than 500 bp with a 38.35% G + C content. A total of 5721 coding sequences were predicted. The genome sequencing information from strain XG196 was submitted to the National Center for Biotechnology Information (NCBI) genome database and the accession number is JABWSY000000000. Nitrate reduction-related genes were annotated in the XG196 genome, including for nitrate reductase (*napA* and *napB*), copper-containing nitrite reductase (*aniA*) and nitrous-oxide reductase (*nosZ*), while the gene encoding nitric oxide reductase (*nor*) was missing. Some assimilatory nitrate reduction-related genes (*nasC*, *nasD*, and *nasE*) were also present in the genome. Genes encoding the molybdate ABC transport system (*modA* and *modB*) were also identified. The 16S rDNA sequence (1487 bp) of strain XG196 identified the organism as a member of the *Bacillus* genus. In order to characterize it at the species level, a total of 190 16S rDNA sequences, which include those of two XG196 close relatives, 186 *Bacillus* type strains and one out group strain, were used to build a phylogenetic tree using maximum likelihood by IQ-TREE (Supplementary Figure S3). Strain XG196 is closely related to *B. niacini* RB-113 (non-type, 99.176%), *B.* sp. LMG20241 (non-type, 99.663%), *B. niacini* IFO15566 (type, 99.193%), and *B. drentensis* LMG 21831 (type, 99.084%).

### Phylogenetic Analysis of the Molybdate Binding Protein (ModA) of XG196

**FIGURE 3:**
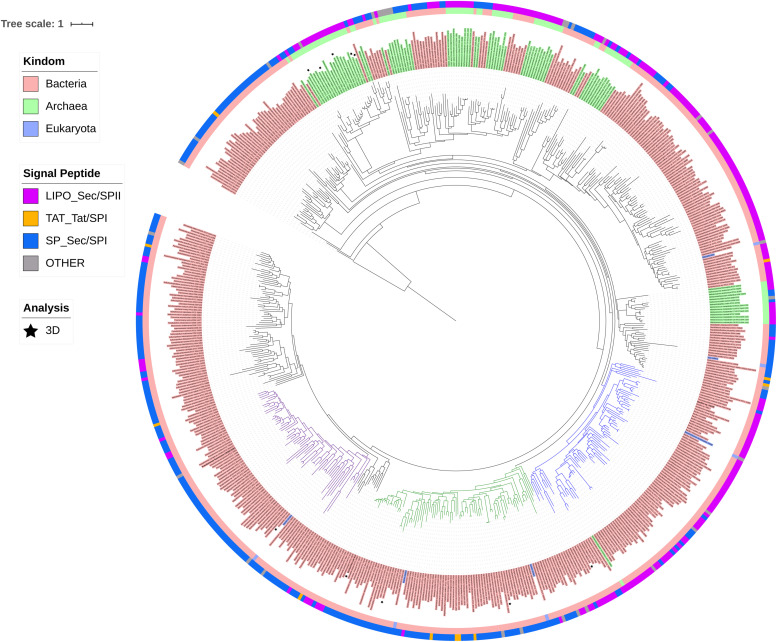
Phylogenetic analysis of ModA. Rooted phylogenetic tree of ModA and WtpA from Bacteria (pink, inner circle), Archaea (green, inner circle), and Eukaryota (blue, inner circle). Outer circle indicates different signal peptide type (LIPO_Sec/SPII in purple, TAT-Tat/SPI in yellow, SP_Sec/SPI in blue and other in gray). ModA proteins with PDB 3D structure data were indicated as black stars. Clades where XG196 ModA (blue clade), N2E2 ModA (purple clade), and *E. coli* ModA (green clade) belong to were also labeled in different colors.

**FIGURE 4:**
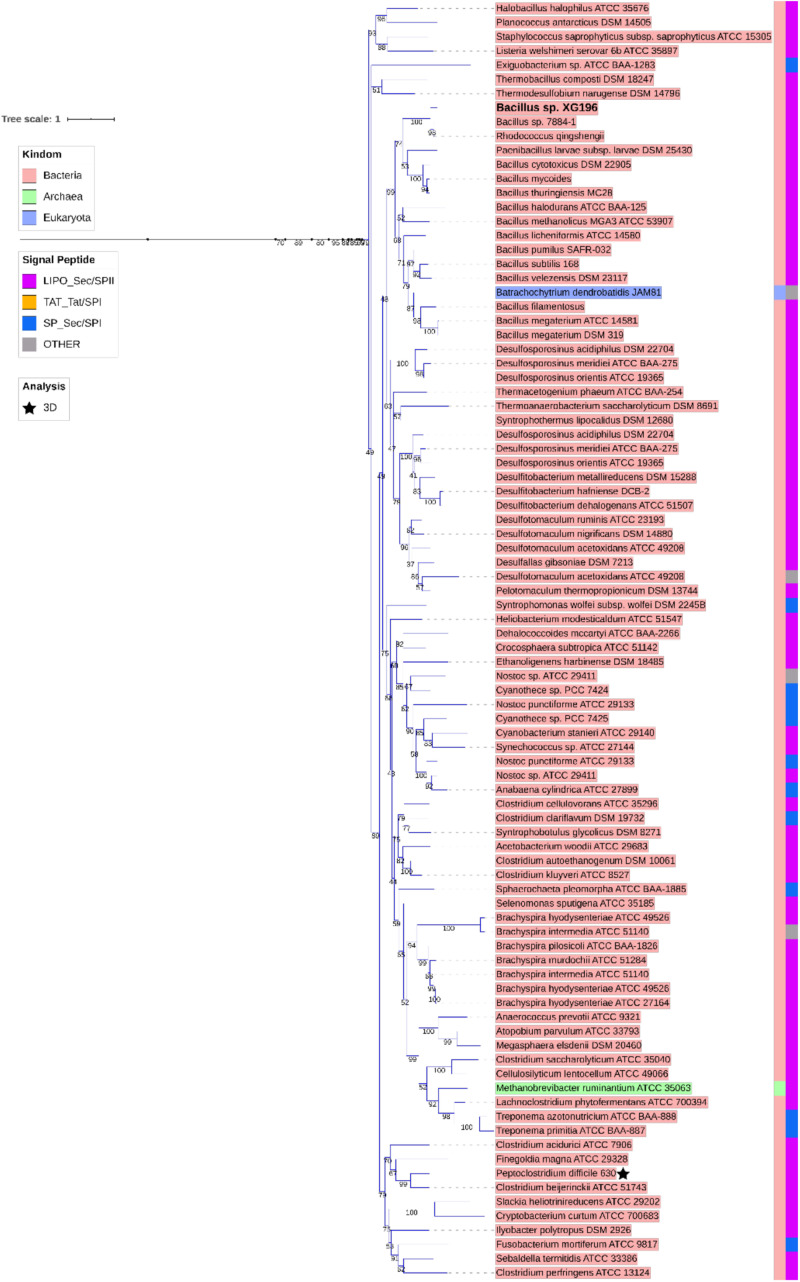
Zoomed-in clade of XG196 ModA and its close relatives. Bootstrap data are shown in the middle of each branch.

ModA is the molybdate-binding protein component of the molybdate ModABC transporter. We hypothesize that the ability of XG196 to grow by nitrate reduction using an extremely low concentration of Mo [<1 nM in contaminated groundwater close to S-3 ponds area (Ge et al., 2019)] is because its ModA has an unusually high affinity for molybdate. Phylogenetic analysis of XG196 ModA based on protein sequences of about 600 ATCC and DSM strains, including those from Archaea, Bacteria and Eukaryota, showed that it is, indeed, distinct from those of the ORR isolate *P*. *fluorescens* N2E2 (N2E2) and of *E. coli* K12 (Figure 3). The same conclusion was reached by a similar analysis using over 4,000 strains (Supplementary Figure S4). Most ModA proteins in proximity to XG196 ModA on the phylogenetic tree originate from other *Bacillus* strains, most of which were also isolated from soil, but their sequence identities are only about 50% (Supplementary Table S4). The two closest relatives of XG196 ModA are from *Rhodococcus qingshengii* (entry: A0A4R6A6K9, 85.9% identity) and *Bacillus* sp. 7884-1 (entry: A0A268JZS1, 85.9% identity) by UniProt BLAST (Figure 4 and Supplementary Table S4).

**FIGURE 5:**
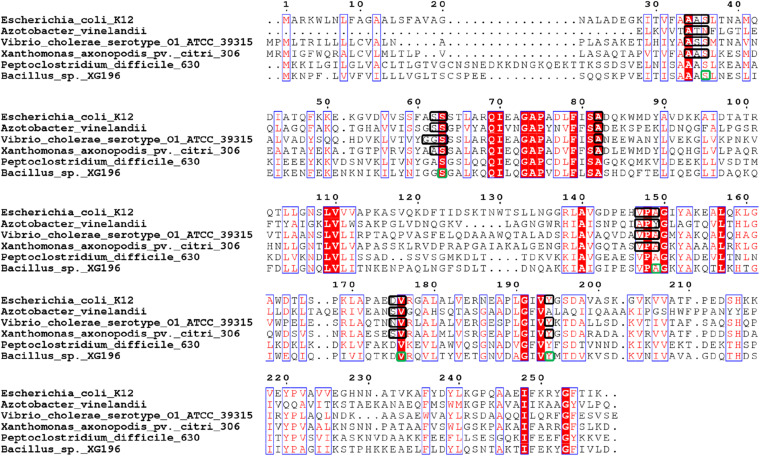
Multi-alignment analysis of *Peptoclostridium difficile* 630 (ModA, UniProt Entry: Q18A64, PDB: 4KD5), *Xanthomonas axonopodis* pv. *citri* 306 (ModA, UniProt Entry: Q8PHA1, PDB: 2H5Y, ${\text{MoO}}_{4}^{2-}$), *Escherichia coli* K12 (ModA, UniProt Entry: P37329, PDB: 1AMF, ${\text{MoO}}_{4}^{2-}$), *Vibrio cholerae* serotype O1 ATCC 39315 (ModA, UniProt Entry: Q9KLL7, PDB:4RXL, ${\text{WO}}_{4}^{2-}$), *Azotobacter vinelandii* (ModA, UniProt Entry: Q7SIH2, PDB: 1ATG, ${\text{WO}}_{4}^{2-}$) and *Bacillus* sp. XG196. Identical residues are in white and boxed in red, while similar residues are in red and boxed in blue. Molybdate/tungstate binding residues of ModA proteins resolved by crystal structures were indicated in black squares. Molybdate binding residues of XG196 ModA identified by protein modeling using *X. axonopodis* pv. *citri* 306 ModA (PDB: 2H5Y) are indicated in green squares.

The structures of the ModA proteins of *Peptoclostridium difficile* 630, also known as *Clostridium difficile* 630 (PDB: 4KD5), *X. axonopodis* pv. *citri* 306 (PDB: 2H5Y, ${\text{MoO}}_{4}^{2-}$), *E. coli* K12 (PDB: 1AMF, ${\text{MoO}}_{4}^{2-}$), *Vibrio cholerae* serotype O1 ATCC 39315 (PDB:4RXL, ${\text{WO}}_{4}^{2-}$), and *Azotobacter vinelandii* (PDB: 1ATG, ${\text{WO}}_{4}^{2-}$) have been determined (Figures 3, 5) (Hu et al., 1997; Lawson et al., 1998; Santacruz et al., 2006). Each binds a single molybdate (or tungstate) ion. In addition, some archaea are able to utilize tungsten, a metal seldom used in biology, in their pyranopterin-containing enzymes (other than Mo-dependent nitrate reductase) (Cabello et al., 2004; Bevers et al., 2006). These tungsten-utilizing microorganisms take up tungstate using a transporter (WtpA) that is highly homologous to ModA (Supplementary Figure S5), and the structures of WtpA from *Methanosarcina acetivorans* ATCC 35395 (PDB: 3CFX, ${\text{WO}}_{4}^{2-}$), *Methanocaldococcus jannaschii* ATCC 43067 (PDB: 3CFZ, ${\text{WO}}_{4}^{2-}$), *Pyrococcus furiosus* ATCC 43587 (PDB: 3CG1, ${\text{WO}}_{4}^{2-}$), *Archaeoglobus fulgidus* ATCC 49558 (PDB: 3CIJ, ${\text{WO}}_{4}^{2-}$) and *P. horikoshii* ATCC 700860 (PDB: 3CG3, ${\text{WO}}_{4}^{2-}$) are known, all of which bind one tungstate ion (Supplementary Figure S5) (Hollenstein et al., 2009).

XG196 ModA was modeled using ModA (PDB: 2H5Y) from *X. axonopodis* pv. *citri* 306 as the template, which has 37% sequence identity with XG196 ModA and contains molybdate as the ligand in the crystal structure. Based on the residues invoved in molybdate binding in *X. axonopodis* pv. *citri* 306 ModA, Ser36, Ser63, Ala149, Val176, and Tyr194 of XG196 ModA are predicted to directly bind molybdate via hydrogen bonds (Figure 5 and Supplementary Figure S6). However, from the modeling it is not clear why the XG196 protein has increased affinity for the metal. Although archaeal and bacterial WtpA/ModA proteins are evolutionally distant, the residues involved in metal binding are partially conserved, suggesting a similar ligand binding mechanism (Figure 3 and Supplementary Figure S5). Multi-alignment analysis of the ModA proteins from *E. coli* K12, XG196, two close relatives of XG196 ModA (from *Rhodococcus qingshengii* and *Bacillus* sp. 7884-1), other *Bacillus* ModA proteins from phylogenetic analysis (Figure 3 and Supplementary Table S4) and of EB-106 isolate XG77, isolated from sediments of similar depth with isolate XG196 (Figures 1A,B), are shown in Supplementary Figure S7. The mean sequence identity and similarity of these ModA sequences is about 12.2 and 65.4%, respectively. The sizes of these ModA proteins are similar (about 250 residues) and their sequences are conserved at 11 out of 12 the molybdate binding residues found in *E. coli* K12 ModA (Ala34, Ala35, Ser36, Ser63, Ala82, Val147, Pro148, Ala149, Asp175, Val176, and Tyr/Phe194), the exception being position Ser/Gly/Ala62. It seems that ModA proteins are quite similar, particularly XG196 ModA and other *Bacillus* ModA proteins, and novel attributes of the XG196 protein are not obvious, especially in the deduced oxyanion binding site.

### ITC Analysis of ModA Proteins

To determine their molybdate-binding properties, the genes encoding the ModA proteins from XG196, N2E2 and *E. coli* were expressed in, and the recombinant proteins were purified from, *E. coli*. ICP-MS analysis showed that XG196 ModA(40 μM) can naturally bind about 67 nM of Mo even when trace grade chemicals were used, higher than what N2E2 ModA (15 nM) and *E. coli* ModA (9 nM) can bind (Supplementary Figure S8). After dialysis in low salt ITC buffer, all ModA proteins can pick up a little bit more Mo from the ITC buffer (XG196 ModA to 82 nM, N2E2 ModA 19 nM, and *E. coli* ModA 10 nM). On average, XG196 ModA, N2E2 ModA and *E. coli* ModA bound 0.002, 0.0005, and 0.0003 of molybdate per protein, respectively, which are far away from being saturated. ITC analysis showed that these proteins contain a single binding site for molybdate (values were 1.10 ± 0.01, 0.95 ± 0.08, and 0.92 ± 0.01, respectively). However, the molybdate binding curves showed that XG196 ModA had a K_*D*_ value for molybdate of 2.21 ± 1.03 nM, which is about one order of magnitude lower than those of N2E2 (27.0 ± 6.2 nM) and *E. coli* (25.01 ± 3.7 nM) ModA (Table 1 and Supplementary Figure S9). Hence, XG196 ModA has a much higher affinity for molybdate, consistent with results from the physiological study showing that XG196 is able to grow by nitrate reduction using Mo concentrations (<1 nM) that limit the growth of other bacteria, including N2E2. The tungstate-binding affinity of XG196 ModA was about fivefold higher than that for molybdate (K_*D*_ 11.15 ± 1.34 nM), and about half of the tungstate dissociation constent values for the ModA proteins of N2E2 and *E. coli* (26.6 ± 2.0 and 23.7 ± 0.6 nM, respectively; see Table 1 and Supplementary Figure S9). The stoichiometry of tungstate binding to each of these proteins was also 1:1, as found for molybdate. Hence, the lower binding affinity for molybdate than tungstate of XG196 ModA is consistent with the better growth of the organism under nitrate-reducing conditions than N2E2 when tungstate is present (Figure 2B).

**TABLE 1:** Molybdate and tungstate binding properties of ModA proteins determined by isothermal titration calorimetry and displacement titration.

ModA	Molybdate			Tungstate		
	K_*D*_ (nM)	N^*a*^	ΔH (kcal/mol)	K_*D*_ (nM)	N	ΔH (kcal/mol)
XG196	2.2 ± 1.0	1.1 ± 0.0	−4.0 ± 0.3	11.2 ± 1.3	1.1 ± 0.1	−4.2 ± 0.3
	2.0 ± 0.2^*b*^	0.8 ± 0.0^*b*^	−3.8 ± 0.5^*b*^	10.6 ± 2.7^*b*^	0.9 ± 0.0^*b*^	−4.0 ± 0.1^*b*^
N2E2	27.0 ± 6.2	1.0 ± 0.1	−5.0 ± 0.3	26.7 ± 2.1	0.9 ± 0.1	−4.4 ± 0.3
*E. coli*	25.0 ± 3.7	0.9 ± 0.0	−5.9 ± 0.0	23.8 ± 0.6	0.9 ± 0.0	−4.9 ± 0.2

*^a^N = measured stoichiometry (oxyanion per protein). ^b^Data obtained by displacement titration using chromate ($CrO_{4}^{2-}$) as the weak binding ligand.*

The K_*D*_ value of XG196 ModA for molybdate was extremely low (≤2 nM), which is only just within the confidence range of the direct ITC approach. Hence, another approach known as displacement titration analysis was used (Sigurskjold, 2000; Krainer and Keller, 2015). Chromate (${\text{CrO}}_{4}^{2-}$) was use as the weak binding ligand. ITC analysis of XG196 ModA binding chromate gave the following results: K_*D*_ = 1.56 ± 0.05 μM, *N* = 0.83 ± 0.09, and ΔH = −1.71 ± 0.26 kcal/mol. XG196 ModA saturated with chromate was then titrated with molybdate or tungstate. The results for molybdate (K_*D*_ = 2.04 ± 0.19, *N* = 0.84 ± 0.02, ΔH = −3.76 ± 0.49 kcal/mol) and tungstate (K_*D*_ = 10.6 ± 2.6, *N* = 0.87 ± 0.02, ΔH = −4.01 ± 0.49 kcal/mol), are similar to those obtained using ITC analysis of direct molybdate or tungstate titrations (Table 1).

### Gene Abundance of Mo-Related Proteins in ORR Groundwater

To better understand the utilization of Mo in the ORR environment, the abundances of ModA genes and genes encoding representative proteins from the four families of Mo proteins were analyzed in ORR groundwater samples from both contaminated and background wells. As shown in Table 2, the abundance of Mo-related genes are generally higher in ORR contaminated groundwater samples. In particular, the abundance of *modA* (encoding ModA) and *napA*/*narG* (encoding dissimilatory nitrate reductase Mo-containing subunit) are significantly higher in contaminated well FW021, FW104 and FW106 (*modA* 27 to 39.9 copies per 10^8^ reads, *napA* 11.2 to 32.5 copies per 10^8^ reads, and *narG* 26.4 to 42.6 copies per 10^8^ reads) than in background well FW300, FW301, and FW305 (*modA* 4.1 to 27 copies per 10^8^ reads, *napA* 1.6 to 8.9 copies per 10^8^ reads, and *narG* 1.8 to 10.5 copies per 10^8^ reads). In contrast, the abundance of *nasA* (encoding assimilatory nitrate reductase Mo-containing subunit), *dmsA* (encoding DMSO reductase Mo-containing subunit), *xdhB* (xanthine oxidase/dehydrogenase) and *sorA* (encoding sulfite oxidoreductase Mo-containing subunit) were only slightly higher in contaminated wells, while the abundance of *nifK* (encoding nitrogenase) is similar in both contaminated and background wells (Table 2). The higher abundance of *modA*, *napA*, and *narG* relative to other Mo-related protein genes in the contaminated wells is likely an adaptive advantage given the high nitrate concentrations (0.02–13.3 mM), which are about 1000-fold higher than in the background wells (0.1–1.8 μM).

**TABLE 2:**
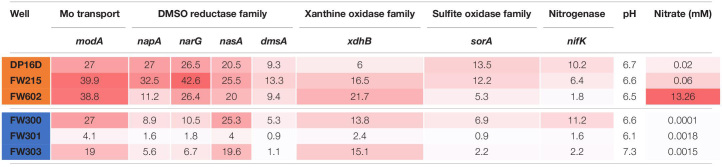
Gene abundance of Mo-related proteins in ORR groundwater.

*The values represent gene copies per 10^8^ reads in samples from contaminated wells (orange) and background wells (blue). The pH and nitrate concentration of each sample is also indicated. The higher values for gene abundance and nitrate are indicated in red.*

## Discussion

The ORR S-3 ponds contamination plume is unique as it contains high concentrations of nitrate (up to 230 mM in groundwater) and various metals (Cd, Ni, Cu, Co, Mn, U, etc.) at low pH (∼3) (Brooks, 2001; Revil et al., 2013). Yet, we previously showed that in this unique environment, Mo is generally limiting for microbial nitrate reduction (Thorgersen et al., 2015). Previous studies have revealed that complex microbial communities survive in this contaminated site (Abulencia et al., 2006; Vishnivetskaya et al., 2011). The overall goal of this research was to elucidate the molecular mechanisms that give certain microorganisms competitive advantages in these extreme habitats. *Bacillus* strain XG196 was isolated from contaminated core EB-106 that was drilled adjacent to the origin of the contamination (the S-3 ponds). XG196 was shown to grow by nitrate reduction in the presence of an exceedingly low concentration of Mo that contaminated its defined medium from the inoculum and the chemicals that make the media (to which no Mo was added). The ability to grow with limited Mo appears to be due to its molybdate-binding protein, ModA, which has a very high affinity for molybdate (K_*D*_ ∼ 2 nM). This is the lowest K_*D*_ value yet reported for any ModA to date and it is also the first ModA characterized from a *Bacillus* strain. Previous studies have typically reported molybdate affinities with ModA proteins that are more than an order of magnitude lower (Corcuera et al., 1993; Bevers et al., 2006; Smart et al., 2009; Aryal et al., 2012). A similarly high affinity but for tungstate was reported for the Wtp protein of W-dependent *P. furiosus*, a member of the archaea domain. Its binding affinity of molybdate is about fivefold lower (K_*D*_ = 11 ± 5 nM) than that found here for XG196 ModA (Bevers et al., 2006).

The molybdate binding affinity of *E. coli* ModA measured in this study (∼25 nM) is consistent with what has been reported by others (K_*D*_ = 20–26 nM; Corcuera et al., 1993; Imperial et al., 1998). The K_*D*_ value for molybdate of N2E2 ModA is about 27 nM, consistent with the poor nitrate-reducing growth observed in Mo-limited media compared to XG196. In addition, the ModA proteins of *E. coli* and N2E2 have very similar K_*D*_ values for both molybdate and tungstate, hence, neither protein is able to distinguish between these two oxyanions, consistent with what has been reported for *E coli* ModA (Rech et al., 1996; Imperial et al., 1998). In contrast, XG196 ModA has a fivefold higher affinity for molybdate compared to tungstate, which could give the organism a selective advantage in scavenging molybdate for growth in the presence of tungstate as seen in the Mo/W competition growth studies herein (Figure 2).

Phylogenetic analysis showed that XG196 ModA is distinct from previously described ModA proteins, including that of *E. coli* K12 (Corcuera et al., 1993; Aryal et al., 2012), the WtpA/ModA proteins from the bacterium *Azotobacter vinelandii* (Lawson et al., 1998), and the archaea *P. horikoshii* (Hollenstein et al., 2009) and *P. furiosus* (Bevers et al., 2006). Multi-alignment analysis indicates that XG196 ModA is quite similar to those of other *Bacillus* species based on their sequence (65.4% mean similarity) and their deduced oxyanion binding sites (Supplementary Figure S7). However, it is hard to conclude that all of these *Bacillus* ModA proteins have molybdate affinities as high as that of XG196 ModA since the molybdate-binding residues are highly conserved. Unfortunately, modeling of XG196 ModA (Supplementary Figure S6) did not shed light on why it has a much higher affinity for molybdate than structurally-characterized proteins. ModA proteins contain a signal peptide at the N terminus that enables the protein to be transported across the membrane. ModA signal peptides fall into one of four different groups: Sec/SPI, Sec/SPII, Tat/SPI, and other (Nielsen et al., 2019). Surprisingly, the ModA from XG196 grouped with the ModA proteins from archaea, and these are all predicted to be lipopeptides and belong to the Sec/SPII group, while N2E2 and *E. coli* ModA proteins belong to the Sec/SPI group with non-lipopeptides (Figure 3). Substrate-binding lipoproteins are widely observed in gram-positive bacteria (Sutcliffe and Russell, 1995; Hutchings et al., 2009). It is believed that the lipopeptides can tether substrate-binding proteins in order to prevent their loss into the growth environment because of the absence of the retentive outer membrane in gram-positive bacteria (Sutcliffe and Russell, 1995). At present, not enough information is available to distinguish “high” affinity molybdate transporters (like XG196 ModA) from “low” affinity ones (like those of N2E2 and *E. coli* ModA) based only on sequence similarity or the deduced molybdate-binding residues. Structural determinations of high affinity ModA proteins in addition to that of XG196 will be required to elucidate the molecular basis as to why these particular proteins bind molybdate so tightly.

Strain XG196 exhibited higher nitrate reductase specific activity than the other EB-106 strains XG95, XG146, and XG201 (Supplementary Figure S1) and accumulated more Mo in its cytoplasm than XG77, XG95, XG146, and XG201 (Supplementary Figure S10). This could be the result of the higher molybdate affinity of its ModA, which must provide more than sufficient Mo for the biosynthesis of functional pyranopterin cofactor in nitrate reductase (Schwarz and Mendel, 2006) when Mo is limited in the environment. XG196 also accumulated the second highest concentration of Mo in the membrane fractions compared to the other EB-106 strains, which might be the result of a high nitrate reductase concentration in the membrane because of more than sufficient Mo taken up from environment. However, these results might not be directly related to the high affinity of ModA for molybdate. Nitrate reductases with high specific activities or high affinity molybdate storage proteins described in previous studies (Pienkos and Brill, 1981; Grunden and Shanmugam, 1997) could also contribute to XG196 being able to grow robustly under nitrate reducing conditions with limited Mo. Further study is required to clarify this issue.

Mo is removed from groundwater in the ORR contaminated area but not from the non-contaminated area as a result of Fe and Al precipitation (Ge et al., 2019). The low Mo concentrations (picomolar range) in the ORR contaminated environment is unusual but not unique. Low Mo concentrations (5–70 nM) occur in naturally-acidic groundwater (pH 2.4 to 2.9) (Nordstrom, 2015), in an acid mine drainage (<10 nM) (Sánchez-España et al., 2016), in harbors (<20 nM) as a result of sedimentary processes (Morford et al., 2007), and in various aquifers, including the Yorkshire Chalk aquifer (<10 nM) due to co-precipitation with or adsorption to sulfide minerals under strong reducing conditions (Smedley et al., 2014). These environments have significantly lower Mo concentrations than most freshwater and open seawater systems, which are typically > 300 nM (Smedley and Kinniburgh, 2017). Limiting Mo concentrations in natural water systems could lead to other environmental problems, for example, by affecting critical steps in the nitrogen cycle, such as nitrate reduction, leading to nitrate accumulation or to slowing down of nitrate removal from contaminated water or soil systems.

There are several factors that affect nitrate reduction in the ORR contaminated environment besides lack of the essential metal Mo. These include the acidic conditions, high nitrate concentrations, the presence of heavy metal contaminants, and limited availability of carbon sources to serve as electron donors for nitrate reduction (Smith et al., 2015; Thorgersen et al., 2015; Ge et al., 2019). Other factors, such as O_2_ concentrations in the soil and groundwater (Zumft, 1997; Qu et al., 2016), temperature and denitrifier community composition (Wallenstein et al., 2006), can also affect the efficiency of nitrate reduction. Meanwhile, the higher abundance of genes encoding the molybdate transport protein (modA) and assimilatory nitrate reductase Mo-containing subunits (*napA*/*narG*) in nitrate-contaminated wells indicates enhanced nitrate reduction in the ORR contaminated groundwater. The high abundance of *modA* could result in a greater uptake of molybdate into cells for the biosynthesis of dissimilatory nitrate reductase, enabling microorganisms to survive in the nitrate-contaminated and Mo-limited ORR environment. These numerous complex environmental factors make it difficult to study the relationships between nitrate reduction and natural microbial communities. There are therefore many unanswered questions at present that can be addressed in part by characterizing novel microbial stains with unique molecular mechanisms, as reported here for XG196 and its ModA protein. Such microorganisms could also be instrumental in developing novel methods to remove contaminating nitrate in complex waste environments.

## Data Availability Statement

The datasets presented in this study can be found in online repositories. The names of the repository/repositories and accession number(s) can be found below: “https://www.ncbi.nlm.nih.gov/nuccore/JABWSY000000000.”

## Author Contributions

XG, MT, and FP designed this study, performed the experiments, analyzed and interpreted the data. MA directed the research. AD, J-MC, and PN carried out the genome sequencing. SG-D, LL, and TN performed ORR metagenome annotation and analysis. TH contributed to environmental sampling. XG wrote the manuscript. MT, FP, AD, J-MC, PN, SG-D, PA, AA, TH, and MA contributed to its revision. All authors contributed to the article and approved the submitted version.

## Conflict of Interest

The authors declare that the research was conducted in the absence of any commercial or financial relationships that could be construed as a potential conflict of interest.

## Footnotes

1 https://blast.ncbi.nlm.nih.gov

2 http://www.kbase.us

3 https://blast.ncbi.nlm.nih.gov/Blast.cgi

4 http://rdp.cme.msu.edu/

5 http://www.iqtree.org/

6 https://www.ebi.ac.uk/interpro/

7 http://weizhongli-lab.org/cd-hit/

8 https://www.ebi.ac.uk/Tools/msa/clustalo/

9 http://www.cbs.dtu.dk/services/SignalP/

10 https://www.ebi.ac.uk/Tools/msa/clustalo/

11 https://www.rcsb.org/

12 http://espript.ibcp.fr/ESPript/ESPript/

13 https://swissmodel.expasy.org/

14 http://www.cgl.ucsf.edu/chimera

15 http://www.cbs.dtu.dk/services/SignalP-5.0/

16 https://jgi.doe.gov/data-and-tools/bbtools/
