# Supplementary material for: Characterization of a Metal-Resistant Bacillus Strain With a High Molybdate Affinity ModA From Contaminated Sediments at the Oak Ridge Reservation
Source: Front Microbiol. 2020 Oct 19;11:587127. doi: 10.3389/fmicb.2020.587127 (PMC7604516; doi:10.3389/fmicb.2020.587127)
Supplement: Supplementary file 1 [file Data_Sheet_1.PDF]

Characterization of a metal-resistant *Bacillus* strain with a high molybdate affinity ModA from contaminated sediments at the Oak Ridge Reservation

Xiaoxuan Ge[1], Michael P. Thorgersen[1], Farris L. Poole II[1], Adam M. Deutschbauer[2], John-Marc Chandonia[2], Pavel S. Novichkov[2], Sara Gushgari-Doyle[3], Lauren M. Lui[2], Torben Nielsen[2], Romy Chakraborty[3], Paul D. Adams[4,5], Adam P. Arkin[2,5], Terry C. Hazen[6] and Michael W. W. Adams[1,*]

[1]Department of Biochemistry and Molecular Biology, University of Georgia, Athens, GA, USA
[2]Environmental Genomics and Systems Biology Division, Lawrence Berkeley National Laboratory, Berkeley, CA, USA
[3]Earth and Environmental Sciences, Lawrence Berkeley National Laboratory, Berkeley, CA, United States
[4]Molecular Biosciences and Integrated Bioimaging, Lawrence Berkeley National Laboratory, Berkeley, CA, USA
[5]Department of Bioengineering, University of California, Berkeley, CA, USA
[6]Department of Civil and Environmental Engineering, University of Tennessee, Knoxville, TN 37996, USA

Supplemental Materials

Figures S1-S10 and Tables S1-S5

Figure S1. Nitrate reductase activities of EB-106 isolates.

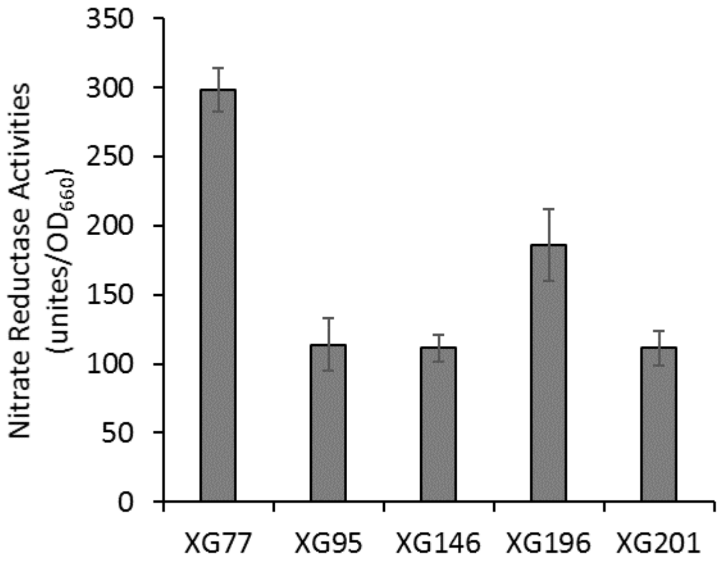

Figure S2. IC_{50} values for individual metals, Metal Mix, nitrate and nitrite for EB-106 strains. Gradient colors from red to green represent lower to higher values.

Strain	Cd^{2+} (μM)	Ni^{2+} (μM)	Cu^{2+} (μM)	Co^{2+} (μM)	Mn^{2+} (μM)	U^{6+} (μM)	Metal Mix (X)	Nitrate (mM)	Nitrite (mM)
XG77	43.33	58.14	70.82	63.72	99.55	>2000	0.69	413.34	167.92
XG146	27.48	69.53	3.94	50.55	>900	>2000	0.422	197.99	71.62
XG95	145.72	74.41	100.35	52.49	>200	>2000	0.39	1326.13	354.02
XG201	50.69	75.62	54.93	129.88	>900	>2000	0.59	478.66	39.84
XG196	23.10	119.45	94.49	220.29	>900	>2000	1.16	299.15	99.01

Figure S3. Rooted phylogenetic tree of 16S rRNA gene sequences from *B. sp.* XG196 and other type *Bacillus* strains. Bootstrap data were labelled at the middle of each branch. Zoomed-in figure shows the close relatives of XG196.

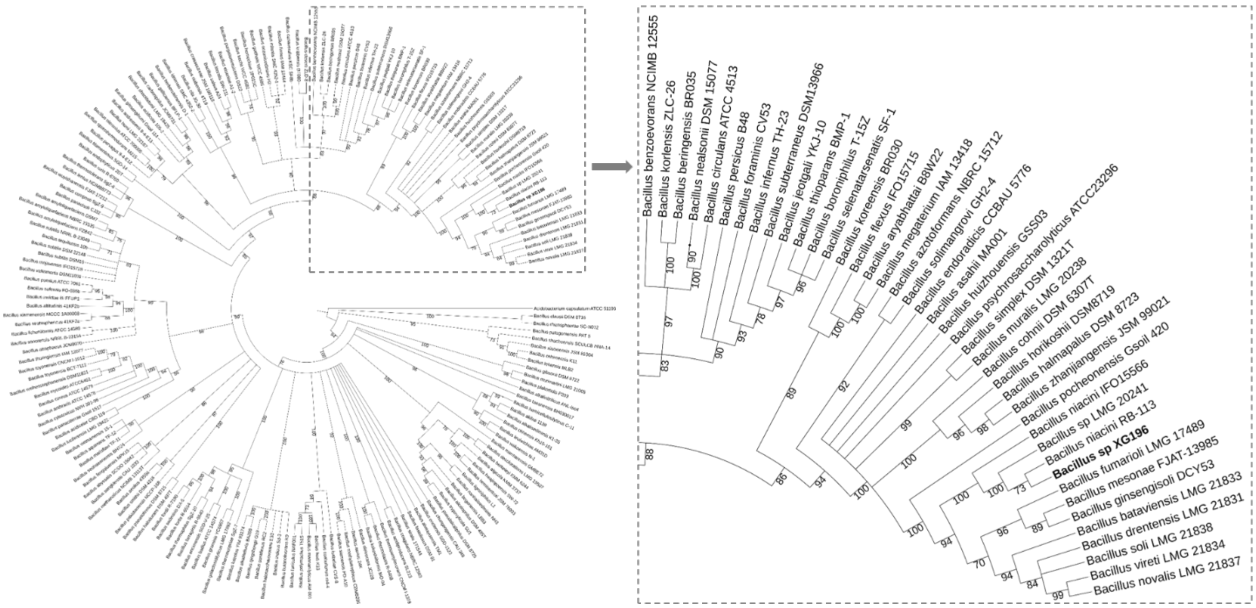

Figure S4. Rooted phylogenetic tree of ModA and WtpA from Bacteria (pink part of circle), Archaea (green part of circle) and Eukaryota (blue part of circle). Clades to which XG196 ModA (blue clade), N2E2 ModA (purple clade) and Ecoli ModA (green clade) belong are labeled in different colors.

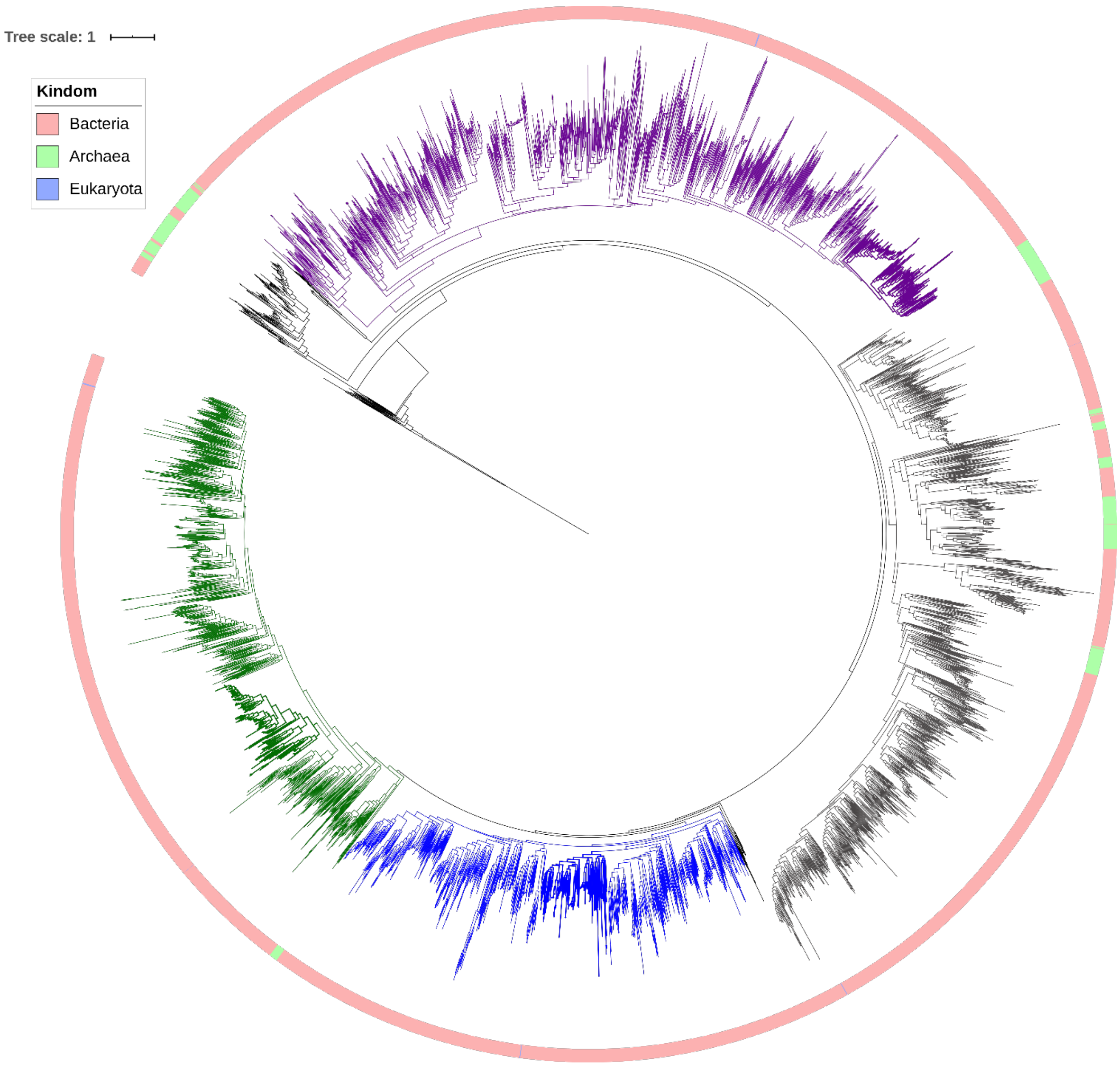

Figure S5. Multi-alignment analysis of ModA/WtpA proteins from *Methanosarcina acetivorans* ATCC 35395 (WtpA/ModA, UniProt Entry: Q8TTZ5, PDB: 3CFX, WO_4^{2-}), *Methanocaldococcus jannaschii* ATCC 43067 (WtpA/ModA, UniProt Entry: Q58586, PDB: 3CFZ, WO_4^{2-}), *Pyrococcus furiosus* ATCC 43587 (WtpA/ModA, UniProt Entry: Q8U4K5, PDB: 3CG1, WO_4^{2-}), *Archaeoglobus fulgidus* ATCC 49558 (WtpA/ModA, UniProt Entry: O30142, PDB: 3CIJ, WO_4^{2-}), *Pyrococcus horikoshii* ATCC 700860 (WtpA/ModA, UniProt Entry: O57890, PDB: 3CG3), *Peptoclostridium difficile* 630 (ModA, UniProt Entry: Q18A64, PDB: 4KD5), *Xanthomonas axonopodis pv. citri* 306 (ModA, UniProt Entry: Q8PHA1, PDB: 2H5Y, MoO_4^{2-}), *Escherichia coli* K12 (ModA, UniProt Entry: P37329, PDB: 1AMF, MoO_4^{2-}), *Vibrio cholerae* serotype O1 ATCC 39315 (ModA, UniProt Entry: Q9KLL7, PDB:4RXL, WO_4^{2-}), *Azotobacter vinelandii* (ModA, UniProt Entry: Q7SIH2, PDB: 1ATG, WO_4^{2-}) and *Bacillus sp.* XG196. Secondary structure of *Pyrococcus furiosus* ATCC 43587 WtpA/ModA (UniProt Entry: Q8U4K5, PDB: 3CG1, WO_4^{2-}) was listed on top. Identical residues are in white and boxed in red, while similar residues are in red and boxed in blue. α- and η-helixes are displayed as squiggles, β-strands are displayed as arrows, strict β-turns are indicated by TT letters. Molybdate/tungstate binding residues of ModA/WtpA proteins resolved by crystal structures were indicated in black squares.

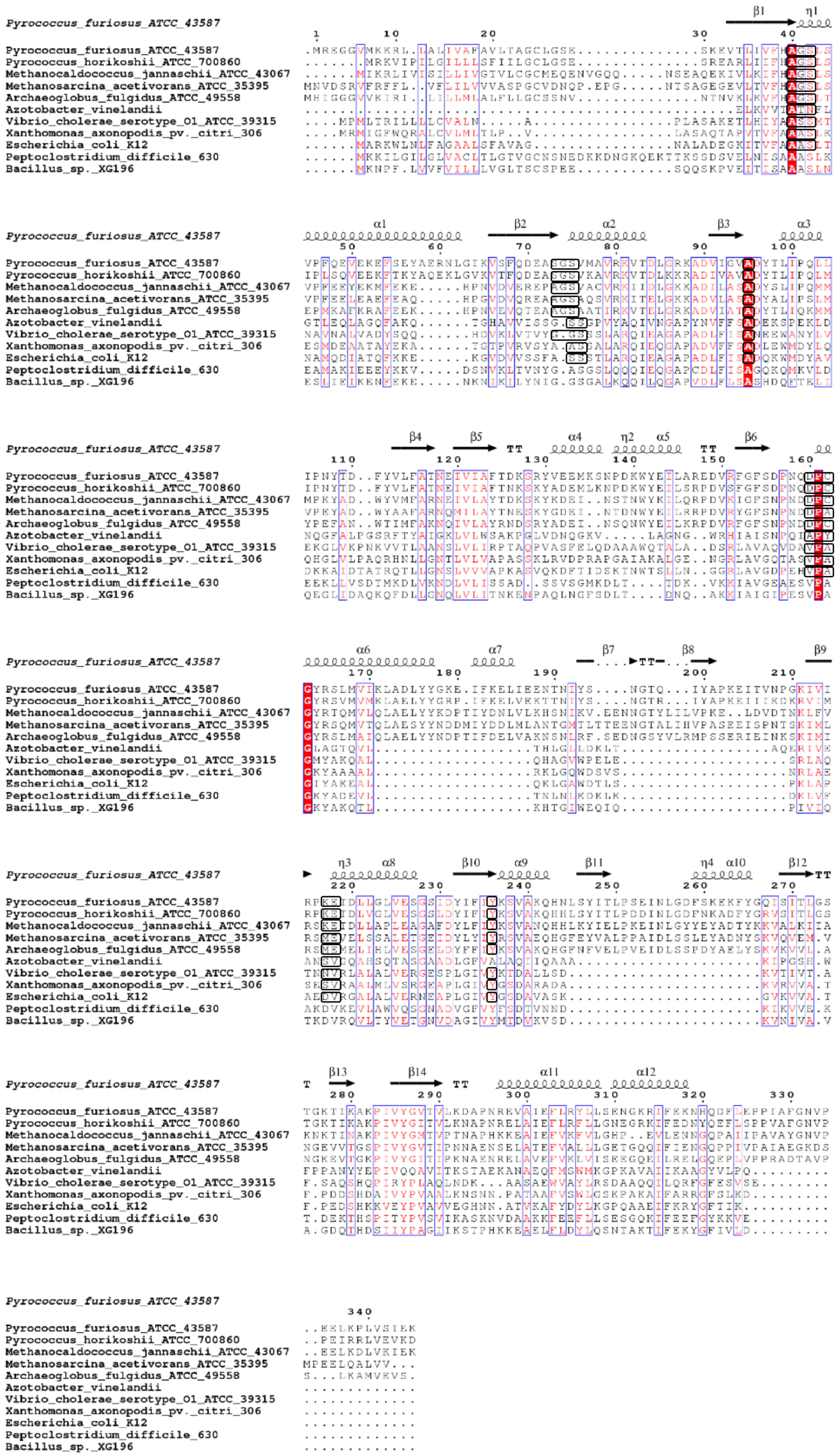
Pyrococcus_furiosus_ATCC_43587
Pyrococcus_horikoshii_ATCC_700860
Methanocaldococcus_jannaschii_ATCC_43067
Methanosarcina_acetivorans_ATCC_35395
Archaeoglobus_fulgidus_ATCC_49558
Azotobacter_vinelandii
Vibrio_cholerae_serotype_O1_ATCC_39315
Xanthomonas_axonopodis_pv._citri_306
Escherichia_coli_K12
Peptoclostridium_difficile_630
Bacillus_sp._XG196

Figure S6. The 3D structure of XG196 ModA was modeled using ModA from *X. axonopodis pv. citri* 306 as the template (PDB: 2H5Y). This has a sequence identity of 36.96 % with XG196 ModA. (A) The overlap of template (PDB: 2H5Y, pink) and XG196 ModA model (tan). Molybdate is labelled in red. (B) Predicted molybdate binding residues in XG196 ModA overlapped with those of the template ModA (PDB: 2H5Y). XG196 ModA residue numbers match the multi-alignments shown in Figure 5. Predicted hydrogen bonds are indicated in yellow lines.

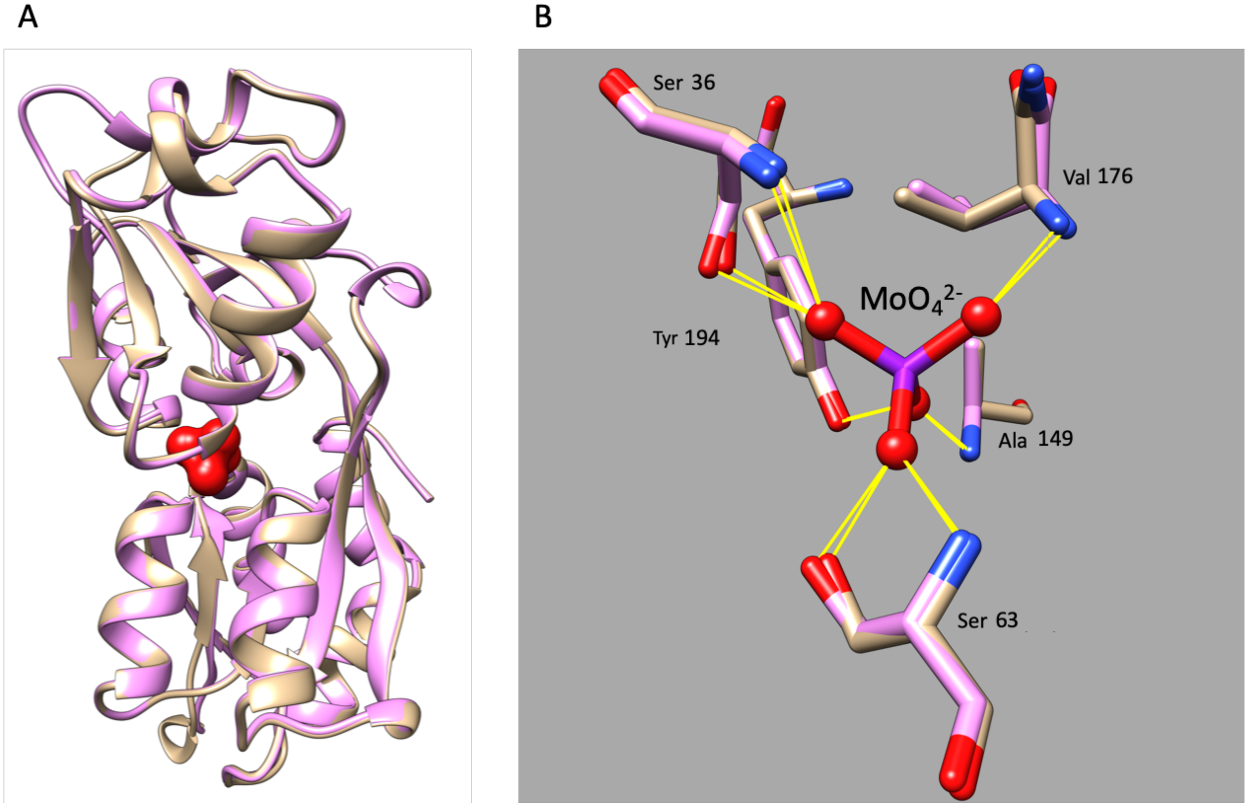

Figure S7. Multi-alignment analysis of ModA proteins from *Escherichia coli* K12 (ModA, UniProt Entry: P37329, PDB: 1AMF, MoO_4^{2-}), EB-106 isolates *Bacillus sp.* XG196 and *Bacillus sp.* XG77 (genome accessible on KBase https://narrative.kbase.us/narrative/61518), two close relatives (*Rhodococcus qingshengii* and *Bacillus sp*. 7884-1) and other *Bacillus* strains from phylogenetic analysis (Figure 3) and listed in Table S4. Secondary structure of *E. coli* K12 ModA was listed on top. Identical residues are in white and boxed in red, while similar residues are in red and boxed in blue. α- and η-helixes are displayed as squiggles, β-strands are displayed as arrows, strict β-turns are indicated by TT letters. Molybdate binding residues of *E. coli* K12 ModA were indicated in black squares.

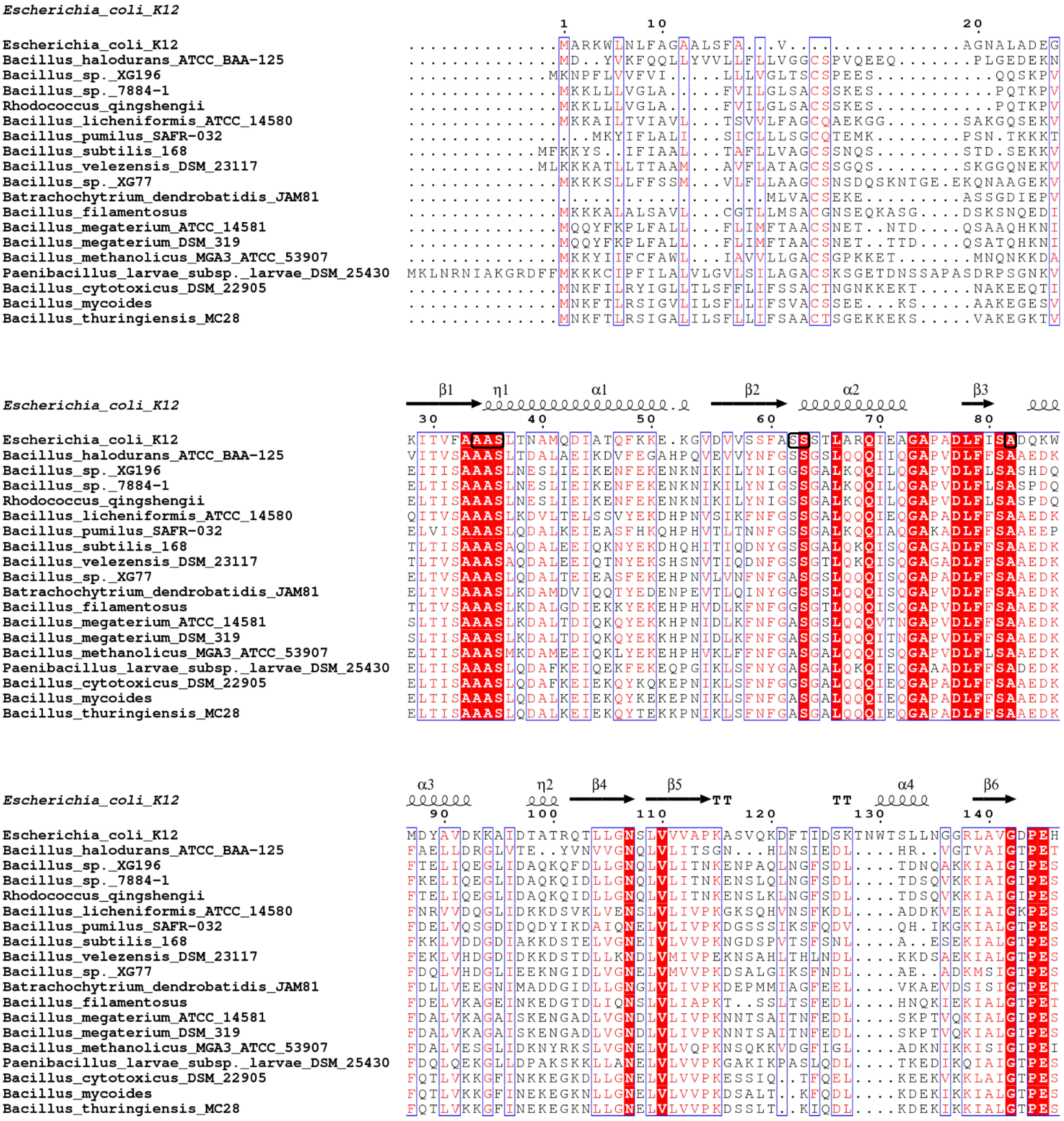

Escherichia_coli_K12
1 10 20
Escherichia_coli_K12 ..............MARKWLNLFAGAALSFA...V...............AGNALADEG
Bacillus_halodurans_ATCC_BAA-125 ..............MD..YVKFQQLLYVVLLFLLVGGCSPVQEEQ.......PLGEDEKN
Bacillus_sp._XG196 .............MKNPFLVVFVI.....LLLVGLTSCSPEES...........QQSKPV
Bacillus_sp._7884-1 ..............MKKLLLVGLA.....FVILGLSACSSKES...........PQTKPV
Rhodococcus_qingshengii ..............MKKLLLVGLA.....FVILGLSACSSKES...........PQTKPV
Bacillus_licheniformis_ATCC_14580 ..............MKKAILTVIAVL...TSVVLFAGCQAEKGG.......SAKGQSEKV
Bacillus_pumilus_SAFR-032 ................MKYIFLALI...SICLLLSGCQTEMK........PSN.TKKKT
Bacillus_subtilis_168 ............MFKKYS..IFIAAL...TAFLLVAGCSSNQS........STD.SEKKV
Bacillus_velezensis_DSM_23117 ............MLKKKATLLTTAAM...AVFLATAGCSSGQS........SKGGQNEKV
Bacillus_sp._XG77 ..............MKKKSLLFFSSM...VLFLLAAGCSNSDQSKNTGE.EKQNAAGEKV
Batrachochytrium_dendrobatidis_JAM81 ....MLVACSEKE.........ASSGDIEPV
Bacillus_filamentosus ..............MKKKALALSAVL...CGTLLMSACGNSEQKASG....DSKSNQEDI
Bacillus_megaterium_ATCC_14581 ..............MQQYFKPLFALL...FLIMFTAACSNET..NTD....QSAAQHKNI
Bacillus_megaterium_DSM_319 ..............MQQYFKPLFALL...FLIMFTAACSNET..TTD....QSATQHKNI
Bacillus_methanolicus_MGA3_ATCC_53907 ..............MKKYIIFCFAWL...IAVVLLGACSGPKKET.......MNQNKKDA
Paenibacillus_larvae_subsp._larvae_DSM_25430 MKLNRNIAKGRDFFMKKKCIPFILALVLGVLSILAGACSKSGETDNSSAPASDRPSGNKV
Bacillus_cytotoxicus_DSM_22905 ..............MNKFILRYIGLLTLSFFLIFSSACTNGNKKEKT.....NAKEEQTI
Bacillus_mycoides ..............MNKFTLRSIGVLILSFLLIFSVACSSEE...KS.....AAKEGESV
Bacillus_thuringiensis_MC28 ..............MNKFTLRSIGALILSFLLIFSAACTSGEKKEKS.....VAKEGKTV
β1 η1 α1 β2 α2 β3
Escherichia_coli_K12
30 40 50 60 70 80
Escherichia_coli_K12 KITVFAAASLTNAMQDIATQFKKE.KGVDVVSSFASSSTLARQIEAGAPADLFISADQKW
Bacillus_halodurans_ATCC_BAA-125 VITVSAAASLTDALAEIKDVFEGAHPQVEVVYNFGSSGSLQQQIIQGAPVDLFFSAAEDK
Bacillus_sp._XG196 EITISAAASLNESLIEIKENFEKENKNIKILYNIGGSGALKQQILQGAPVDLFLSASHDQ
Bacillus_sp._7884-1 ELTISAAASLNESLIEIKENFEKENKNIKILYNIGSSGALKQQILQGAPVDLFLSASPDQ
Rhodococcus_qingshengii ELTISAAASLNESLIEIKENFEKENKNIKILYNIGSSGALKQQILQGAPVDLFLSASPDQ
Bacillus_licheniformis_ATCC_14580 QITVSAAASLKDVLTELSSVYEKDHPNVSIKFNFGSSGALQQQIEQGAPADLFFSAAEDK
Bacillus_pumilus_SAFR-032 ELVISAAASLQDALKEIEASFHKQHPHVTLTNNFGSSGALKQQIAQGAKADLFFSAAEEP
Bacillus_subtilis_168 TLTISAAASAQDALEEIQKNYEKDHQHITIQDNYGSSGALQKQISQGAGADLFFSAAEDK
Bacillus_velezensis_DSM_23117 TLTVSAAASAQDALEEIQTNYEKSHSNVTIQDNFGSSGTLQKQISQGAGADLFFSAAEDK
Bacillus_sp._XG77 ELTVSAAASLQDALTEIEASFEKEHPNVLVNFNFGASGSLQQQISQGAPADLFFSAAEDK
Batrachochytrium_dendrobatidis_JAM81 ELTVSAAASLKDAMDVIQQTYEDENPEVTLQINYGGSGSLQQQISQGAPVDLFFSAAEDK
Bacillus_filamentosus TLTVSAAASLKDALGDIEKKYEKEHPHVDLKFNFGGSGTLQQQISQGAPVDLFFSAAEDK
Bacillus_megaterium_ATCC_14581 SLTISAAASLKDALTDIQKQYEKKHPNIDLKFNFGASGSLQQQVTNGAPVDLFFSAAEDK
Bacillus_megaterium_DSM_319 SLTISAAASLKDALTDIQKQYEKKHPNIDLKFNFGASGSLQQQITNGAPVDLFFSAAEDK
Bacillus_methanolicus_MGA3_ATCC_53907 ELTISAAASMKDAMEEIQKLYEKEHPHVKLFFNFGASGALQQQISQGAPVDLFLSAAEDK
Paenibacillus_larvae_subsp._larvae_DSM_25430 ELTISAAASLQDAFKEIQEKFEKEQPGIKLSFNYGASGALQKQIEQGAAADLFFSADEDK
Bacillus_cytotoxicus_DSM_22905 ELTISAAASLQDAFKEIEKQYKQKEPNIKLSFNFGGSGALQQQIEQGAPADLFFSAAEDK
Bacillus_mycoides ELTISAAASLQDALKEIEKQYKEKEPNIKLSFNFGASGALQQQIEQGAPADLFFSAAEDK
Bacillus_thuringiensis_MC28 ELTISAAASLQDALKEIEKQYTEKKPNIKLSFNFGASGALQQQIEQGAPADLFFSAAEDK
α3 η2 β4 β5 TT TT α4 β6
Escherichia_coli_K12
90 100 110 120 130 140
Escherichia_coli_K12 MDYAVDKKAIDTATRQTLLGNSLVVVAPKASVQKDFTIDSKTNWTSLLNGGRLAVGDPEH
Bacillus_halodurans_ATCC_BAA-125 FAELLDRGLVTE..YVNVVGNQLVLITSGN...HLNSIEDL....HR..VGTVAIGTPET
Bacillus_sp._XG196 FTELIQEGLIDAQKQFDLLGNQLVLITNKENPAQLNGFSDL....TDNQAKKIAIGIPES
Bacillus_sp._7884-1 FKELIQEGLIDAQKQIDLLGNQLVLITNKENSLQLNGFSDL....TDSQVKKIAIGIPES
Rhodococcus_qingshengii FTELIQEGLIDAQKQIDLLGNQLVLITNKENSLKLNGFSDL....TDSQVKKIAIGIPES
Bacillus_licheniformis_ATCC_14580 FNRVVDQGLIDKKDSVKLVENSLVLIVPKGKSQHVNSFKDL....ADDKVEKIAIGKPES
Bacillus_pumilus_SAFR-032 FDELVQSGDIDQDYIKDAIQNELVLIVPKDGSSSIKSFQDV....QH.IKGKIALGTPES
Bacillus_subtilis_168 FKKLVDDGDIAKKDSTELVGNEIVLVVPKNGDSPVTSFSNL....A..ESEKIALGTPES
Bacillus_velezensis_DSM_23117 FEKLVHDGDIDKKDSTDLLKNDLVMIVPEKNSAHLTHLNDL....KKDSAEKIALGTPES
Bacillus_sp._XG77 FDQLVHDGLIEEKNGIDLVGNELVMVVPKDSALGIKSFNDL....AE..ADKMSIGTPES
Batrachochytrium_dendrobatidis_JAM81 FDLLVEEGNIMADDGIDLLGNGLVLIVPKDEPMMIAGFEEL....VKAEVDSISIGTPET
Bacillus_filamentosus FDELVKAGEINKEDGTDLIQNSLVLIAPKT..SSLTSFEDL....HNQKIEKIALGTPET
Bacillus_megaterium_ATCC_14581 FDALVKAGAISKENGADLVGNDLVLIVPKNNTSAITNFEDL....SKPTVQKIALGIPES
Bacillus_megaterium_DSM_319 FDALVKAGAISKENGADLVGNDLVLIVPKNNTSAITNFEDL....SKPTVQKIALGIPES
Bacillus_methanolicus_MGA3_ATCC_53907 FDALVESGLIDKNYRKSLVGNELVLVQPKNSQKKVDGFIGL....ADKNIKKISIGIPEI
Paenibacillus_larvae_subsp._larvae_DSM_25430 FDQLQEKGLLDPAKSKKLLANELVLVVPKGAKIKPASLQDL....TKEDIKQIAIGTPES
Bacillus_cytotoxicus_DSM_22905 FQTLVKKGFINKKEGKDLLGNELVLVVPKESSIQ..TFQEL....KEEKVKKLAIGTPES
Bacillus_mycoides FQTLVKKGFINEKEGKNLLGNELVLVVPKDSALT..KFQDL....KDEKIKKIALGTPES
Bacillus_thuringiensis_MC28 FQTLVKKGFINEKEGKNLLGNELVLVVPKDSSLT..KIQDL....KDEKIKKIALGTPES

Figure S7. Continued

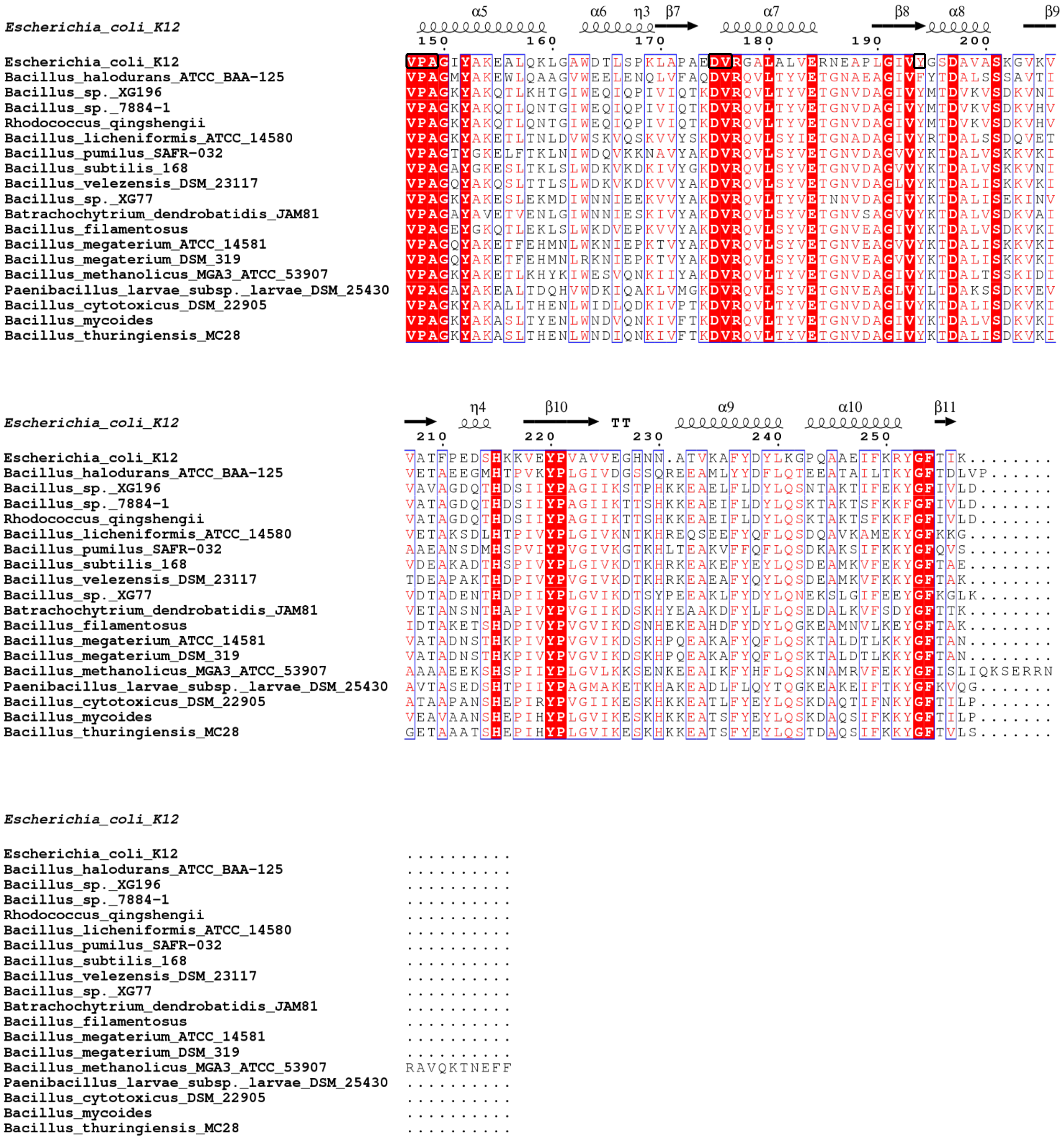

Figure S8. Mo accumulation in the presence of 40 μM molybdate by XG196 ModA, N2E2 ModA or *E. coli* ModA before and after dialysis.

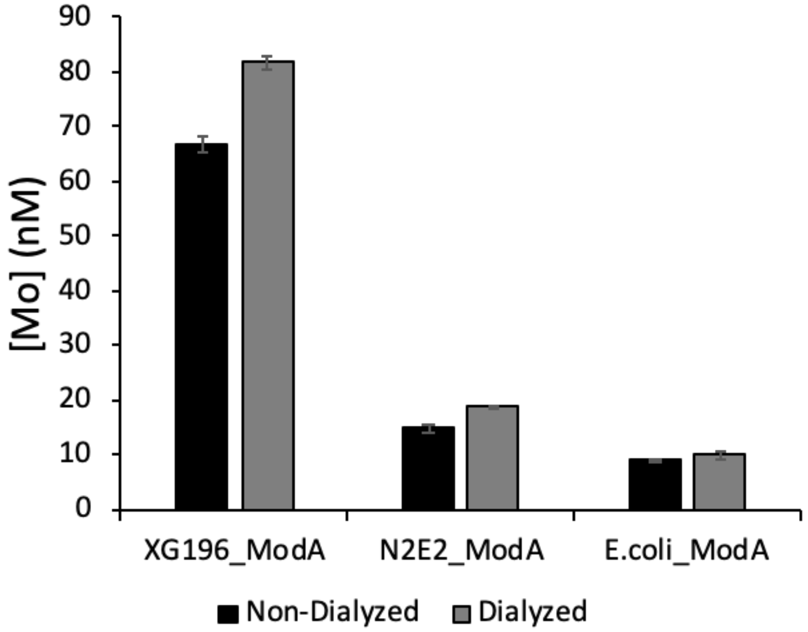

Figure S9. ITC profiles for the binding of molybdate or tungstate by XG196 ModA, N2E2 ModA and *E. coli* ModA.

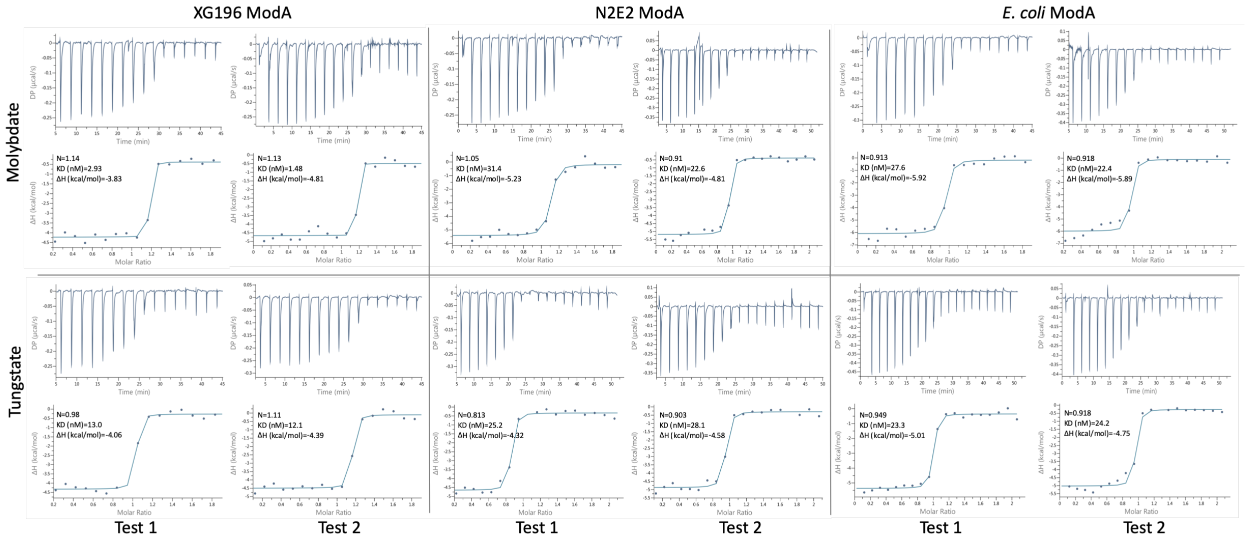

Figure S10. Mo accumulation in ORR isolates under anaerobic nitrate reducing growth using 1 μM molybdate.

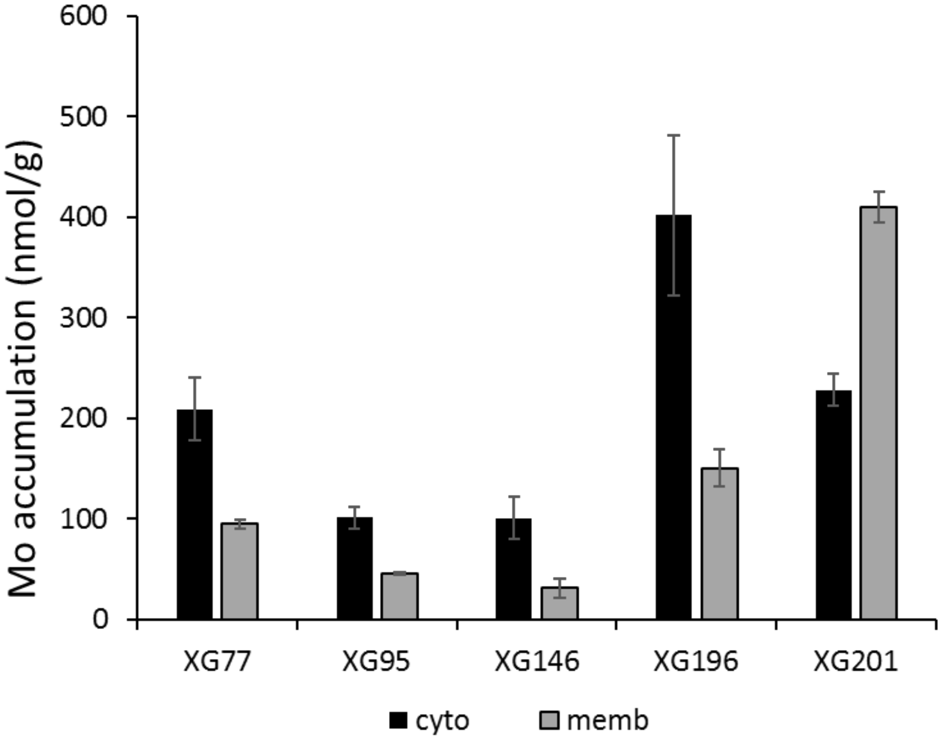

Table S1. Metals used to mimic ORR contamination.

Metal (1 ×)	Compound added	Final Conc. (μM)
Mn^{2+}	$MnCl_2 \cdot 2H_2O$	100
Fe^{2+}	$Fe(NH_4)_2(SO_4)_2 \cdot 6H_2O$	10
Co^{2+}	$CoCl_2 \cdot 6H_2O$	30
Ni^{2+}	$NiCl_2 \cdot 6H_2O$	150
Cu^{2+}	$CuCl_2 \cdot 2H_2O$	10
Cd^{2+}	$Cd(CH_3COO)_2 \cdot 2H_2O$	5
U^{6+}	$UO_2(CH_3COO)_2 \cdot 2H_2O$	100

Table S2. Primers for *modA* amplification.

Primer ID	Primer Sequence	Restriction Enzyme Site
XG196-modA-F	GGAATTC**CATATG**CACCACCACCACCACCACTCGCCCGAAGAATCACAAC	NdeI
XG196-modA-R	CAG**CTCGAG**TCAATCCAATACTATAAAGCC	XhoI
N2E2-modA-F	GGAATTC**CATATG**CACCACCACCACCACCACGAGGTGCAGGTGGCGGTC	NdeI
N2E2-modA-R	GTAT**GGATCC**TTAGCGTTGGTAACCGTAGGCTTGG	BamHI
Ecoli-modA-F	GGAATTC**CATATG**CACCACCACCACCACCACGAAGGGAAAATCACGGTGTTCG	NdeI
Ecoli-modA-R	CAG**CTCGAG**TTACTTGATTGTAAATCCGTAACGTTTAAAG	XhoI

Table S3. Maximum OD_{600} of EB-106 isolates grown on different carbon sources (2 mM) under anaerobic conditions with and without nitrate (20 mM).

Carbon sources	Metabolite class	Growth of XG196		Growth of XG146		Growth of XG201		Growth of XG77		Growth of XG95	
		+ nitrate	- nitrate	+ nitrate	- nitrate	+ nitrate	- nitrate	+ nitrate	- nitrate	+ nitrate	- nitrate
Formate	organic acid	-	-	-	-	-	-	-	-	-	-
Acetate	organic acid	0.11	-	0.11	-	-	-	-	-	-	-
Ethanol	alcohol	-	-	0.1	-	-	-	-	-	-	-
Lactate	organic acid	0.33	-	0.17	-	-	-	0.15	-	-	-
Succinic acid	organic acid	-	-	0.17	-	-	-	0.10	-	0.09	-
Fumarate	organic acid	0.25	-	0.17	-	-	-	0.12	-	0.13	-
Xylose	pentose	0.48	0.13	0.20	-	0.18	-	0.22	-	0.22	-
Xylitol	sugar alcohol	-	-	0.27	-	-	-	-	-	-	-
Glucose	hexose	0.63	0.29	0.27	-	0.23	0.13	0.28	-	0.27	-
Fructose	hexose	0.48	0.22	0.25	-	0.23	0.12	0.25	-	0.27	-
Maltose	dihexose	0.95	0.15	0.37	-	0.38	0.11	0.20	-	0.18	-
Benzoate	aromatic compounds	-	-	-	-	-	-	-	-	-	-
4-hydroxybenzoate	aromatic compounds	-	-	-	-	-	-	-	-	-	-
Tartrate	aromatic compounds	-	-	0.15	-	-	-	-	-	-	-
Proline	amino acid	0.47	0.08	0.19	-	-	-	0.10	-	0.14	-
Phenylalanine	amino acid	-	-	-	-	-	-	-	-	-	-
Arginine	amino acid	0.29	-	0.19	-	-	-	-	-	0.14	-
Threonine	amino acid	-	-	0.15	-	-	-	-	-	-	-
Leucine	amino acid	-	-	-	-	-	-	-	-	-	-
Glutamate	amino acid	0.47	0.11	0.19	-	-	-	0.09	-	0.08	-
Glutamine	amino acid	0.08	-	0.2	-	-	-	0.09	-	0.15	-

* -, no growth ($OD_{600max} \leq 0.07$)

Table S4. ModA proteins in ModA tree closely related to XG196 ModA.

UniProt Entry	ModA Origin Strain	% Identity	Alignment Length	E Value	Bit Score	% positives	Origin/type*
A0A4R6A6K9	*Rhodococcus qingshengii*	85.938	256	3.92E-164	446	93.75	Soil*
A0A268JZS1	*Bacillus sp.* 7884-1	85.938	256	8.91E-164	444	93.36	Unknown
I3E8R5	*Bacillus methanolicus* MGA3 ATCC 53907	52.652	264	1.74E-94	270	71.21	Soil (Heggeset et al., 2012)
C2PQ60	*Bacillus mycoides*	52.344	256	5.41E-93	265	71.88	Soil**
A7GKA9	*Bacillus cytotoxicus* DSM22905	50.373	268	9.43E-92	263	69.4	Food-borne pathogen (Lapidus et al., 2008)
K0FWH0	*Bacillus thuringiensis* MC28	51.55	258	4.89E-91	261	70.16	Forest (Guan et al., 2012)
A0A0B6AUR2	*Bacillus megaterium* ATCC14581	48.473	262	6.41E-90	258	67.94	Soil **
D5DNA1	*Bacillus megaterium* DSM319	48.473	262	3.04E-88	253	67.56	Soil***
V9W849	*Paenibacillus larvae subsp. larvae* DSM25430	50.936	267	9.43E-88	253	64.04	Honey bee pathogen (Djukic et al., 2014)
A7Z8Q5	*Bacillus velezensis* DSM23117	50.202	247	1.01E-87	252	68.42	Soil*
Q65LK2	*Bacillus licheniformis* ATCC14580	49.042	261	4.54E-87	250	65.52	Soil*
A0A1X7EDU8	*Bacillus filamentosus*	46.591	264	2.55E-84	243	67.42	Sediment*
O32208	*Bacillus subtilis* 168	48.016	252	6.04E-84	242	68.25	Lab mutant(Burkholder and Giles Jr, 1947;Zeigler et al., 2008)
F4PF01	*Batrachochytrium dendrobatidis* JAM81	50.413	242	6.34E-84	242	67.36	Frog****
A8FHD9	*Bacillus pumilus* SAFR-032	48.846	260	1.42E-80	234	63.46	Spacecraft assembly clean room (Mohapatra and La Duc, 2012)
Q9K7N2	*Bacillus halodurans* ATCCBAA-125	47.107	242	6.80E-74	217	66.94	Soil*

*Information from https://bacdive.dsmz.de/
** Information from https://microbewiki.kenyon.edu/index.php/MicrobeWiki
*** Information from http://bacmap.wishartlab.com/organisms/1082
**** Eukaryota, information from https://mycocosm.jgi.doe.gov/Batde5/Batde5.home.html

Table S5. Properties of characterized ModA proteins.

**Proteins**	**AA (Putative signal peptide removed)**	**MW (Da) with His_6**	**Putative lipoprotein-attachment site**
XG196 ModA	21-260 (240 AA)	27233.86	Cys20
N2E2 ModA	24-250 (227 AA)	24890.04	none
E.coli ModA	26-257 (232 AA)	25624.07	none

References

Burkholder, P.R., and Giles Jr, N.H. (1947). Induced biochemical mutations in *Bacillus subtilis*. *Am. J. Bot.*, 345-348.

Djukic, M., Brzuszkiewicz, E., Fünfhaus, A., Voss, J., Gollnow, K., Poppinga, L., Liesegang, H., Garcia-Gonzalez, E., Genersch, E., and Daniel, R. (2014). How to kill the honey bee larva: genomic potential and virulence mechanisms of *Paenibacillus larvae*. *PloS one* 9.

Guan, P., Ai, P., Dai, X., Zhang, J., Xu, L., Zhu, J., Li, Q., Deng, Q., Li, S., and Wang, S. (2012). Complete genome sequence of *Bacillus thuringiensis serovar Sichuansis* strain MC28. *J. Bacteriol.* 194**,** 6975-6975.

Heggeset, T.M., Krog, A., Balzer, S., Wentzel, A., Ellingsen, T.E., and Brautaset, T. (2012). Genome sequence of thermotolerant *Bacillus methanolicus*: features and regulation related to methylotrophy and production of L-lysine and L-glutamate from methanol. *Appl. Environ. Microbiol.* 78**,** 5170-5181.

Lapidus, A., Goltsman, E., Auger, S., Galleron, N., Ségurens, B., Dossat, C., Land, M.L., Broussolle, V., Brillard, J., and Guinebretiere, M.-H. (2008). Extending the *Bacillus cereus* group genomics to putative food-borne pathogens of different toxicity. *Chem.-Biol. Interact.* 171**,** 236-249.

Mohapatra, B.R., and La Duc, M.T. (2012). Rapid detection of viable *Bacillus pumilus* SAFR-032 encapsulated spores using novel propidium monoazide-linked fluorescence in situ hybridization. *J. Microbiol. Methods* 90**,** 15-19.

Zeigler, D.R., Prágai, Z., Rodriguez, S., Chevreux, B., Muffler, A., Albert, T., Bai, R., Wyss, M., and Perkins, J.B. (2008). The origins of 168, W23, and other *Bacillus subtilis* legacy strains. *J. Bacteriol.* 190**,** 6983-6995.
